# Regulation Engineering of Alkali Metal Interlayer Pillar in P2-Type Cathode for Ultra-High Rate and Long-Term Cycling Sodium-Ion Batteries

**DOI:** 10.1007/s40820-025-01918-7

**Published:** 2026-01-08

**Authors:** Xu Wang, Zixiang Yang, Yujia Cai, Heng Ma, Jinglei Xu, Rabia Khatoon, Zhizhen Ye, Dashuai Wang, Muhammad Tariq Sajjad, Jianguo Lu

**Affiliations:** 1 State Key Laboratory of Silicon and Advanced Semiconductor Materials, School of Materials Science and Engineering, Zhejiang University, Hangzhou, 310058 People’s Republic of China; 2 Zhejiang HuaDian Electric Equipment Testing and Research Institute Co., Ltd., Hangzhou, 311100 People’s Republic of China; 3 London South Bank University, 103 Borough Road, London, SE1 0AA UK; 4 Institute of Zhejiang University-Quzhou, Quzhou, 324000 People’s Republic of China

**Keywords:** Sodium-ion batteries, Layered oxides, P2-type phase, Dual-site doping, Regulation engineering

## Abstract

Layered oxides have attracted significant attention as cathodes for sodium-ion batteries (SIBs) due to their compositional versatility and tuneable electrochemical performance. However, these materials still face challenges such as structural phase transitions, Na^+^/vacancy ordering, and Jahn–Teller distortion effect, resulting in severe capacity decay and sluggish ion kinetics. We develop a novel Cu/Y dual-doping strategy that leads to the formation of "Na–Y" interlayer aggregates, which act as structural pillars within alkali metal layers, enhancing structural stability and disrupting the ordered arrangement of Na^+^/vacancies. This disruption leads to a unique coexistence of ordered and disordered Na^+^/vacancy states with near-zero strain, which significantly improves Na^+^ diffusion kinetics. This structural innovation not only mitigates the unfavorable P2–O2 phase transition but also facilitates rapid ion transport. As a result, the doped material demonstrates exceptional electrochemical performance, including an ultra-long cycle life of 3000 cycles at 10 C and an outstanding high-rate capability of ~70 mAh g^−1^ at 50 C. The discovery of this novel interlayer pillar, along with its role in modulating Na⁺/vacancy arrangements, provides a fresh perspective on engineering layered oxides. It opens up promising new pathways for the structural design of advanced cathode materials toward efficient, stable, and high-rate SIBs.

**Supplementary Information:**

The online version contains supplementary material available at 10.1007/s40820-025-01918-7.

## Highlights

- A novel “Na–Y” interlayer aggregate is proposed, which acts as a robust interlayer pillar, distinct from previously reported single-ion-based pillar structures.
- The coexistence of ordered and disordered Na⁺/vacancy states resulting from Cu/Y dual-site doping can stimulate rapid Na⁺ diffusion.
- The designed Na_0.67_Y_0.05_Ni_0.18_Cu_0.1_Mn_0.67_O_2_ electrode exhibits outstanding long-term cycling performance (~3000 cycles) and high-rate capability (~ 70 mAh g^−1^ at 50 C).

**Supplementary Information:**

The online version contains supplementary material available at 10.1007/s40820-025-01918-7.

## Graphical abstract

**Figure:**
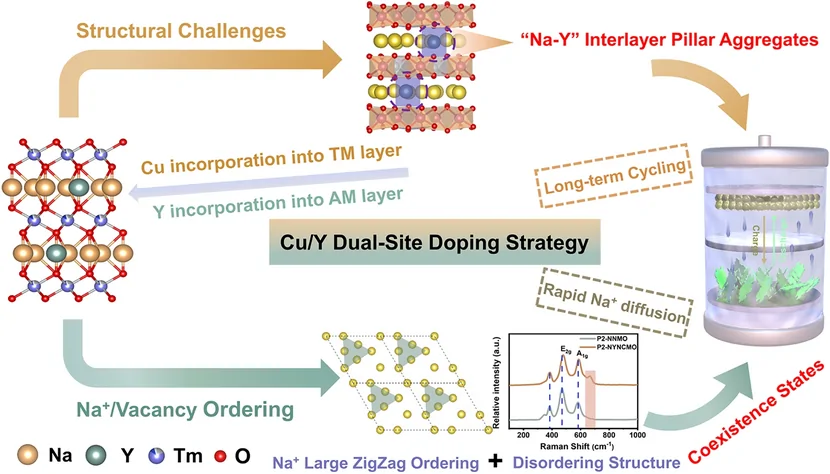

## Introduction

In response to resource constraints, economic concerns, and the growing demand for diversified energy storage, sodium-ion batteries (SIBs) have attracted significant attention in recent years, particularly for large-scale electrical energy storage systems (EESs) due to the natural abundance and low cost of sodium compared to lithium [1–4]. However, SIBs still face challenges such as low energy density and poor cycle life relative to lithium-ion batteries, which hinders their practical application [5–7]. Among the key components influencing the performance of SIBs, the cathode material plays a crucial role, directly affecting both energy density and cycle life [8]. Layered oxides are considered among the most promising cathode materials for SIBs due to their high theoretical capacity, structural and chemical compositional diversity, and compatibility with scalable fabrication processes [9, 10]. Additionally, their structure allows for tunable electrochemical properties through elemental substitution in the host lattice. Layered oxides with the general formula of Na_x_TMO_2_ have two primary phases [11], P2-type [12], and O3-type [13] phases. The symbols "P" or "O" refer to the coordination geometry of the interstitial cations, such as Na^+^ or Li^+^, which are prismatically or octahedrally coordinated with surrounding oxygen ions. The numbers 2 and 3 represent the number of edge-sharing TMO_6_ octahedra with oxygen stacking patterns of ABBA or ABCABC packing, respectively. Among these, the P2-type structure offers superior Na^+^ mobility, and thus better rate capability and cycling stability than O3-type analogs. However, P2-type layered oxides often undergo undesirable structural phase transitions during cycling [14, 15], compromising long-term performance.

To overcome these limitations, various ion-doping strategies have been developed to enhance the structural stability of P2-type materials. This includes substitution with cations such as Mg^2+^ [16], Li^+^ [17, 18], Cu^2+^ [19], Sn^4+^ [20], Al^3+^ [21, 22], Zn^2+^ [23], Nb^5+^ [24] and Ti^4+^ [25, 26], targeting both the transition metal (TM) and alkali metal (AM) layers. In the TM layer, binary [27, 28] and multicomponent substitution [29–31] (e.g., high entropy oxides) is also frequently employed to synergistically optimize the cathode material through utilizing the distinct functions of various elements to suppress structural degradation, increase redox voltage, alleviate effects of Jahn–Teller distortion, and suppress oxygen loss, which are the challenges to be overcome for P2-type layered oxides. The regulation engineering of AM layers typically involves doping with divalent metal ions like Mg^2+^ [32, 33] and Zn^2+^ [34] to shield the electrostatic repulsion between adjacent TMO_2_ layers, especially under conditions of deep sodium extraction. Ahmad et al. [35] exerted a dual-pillar effect to enhance the structural stability by introducing Zn and Mg bimetallic metal ions into the alkali metal layer. This improves structural integrity by limiting interlayer slippage. Additionally, increasing the sodium content in the AM layer has been shown to lower the average oxidation state of TM with more Na^+^ as interlayer pillars, thereby enhancing charge transfer kinetics and preserving structural stability during cycling [36]. Despite these advances, the regulation engineering of AM layers remains a significant challenge. There is currently a lack of clear criteria to evaluate the feasibility and effectiveness of incorporating various metal ions into the AM layers of different host structures. Therefore, further studies are essential to develop a deeper understanding of AM layer regulation and to guide rational design strategies for next-generation SIB cathodes.

Furthermore, the Na^+^ transport mechanism in layered oxide is complex and influenced by many factors, including the Na^+^/vacancy ordering, charge ordering, electron transport, and phase transitions [37, 38]. Among these, the arrangement of Na^+^/vacancy ordering plays a particularly important role in Na^+^ diffusion kinetics. Shi et al. [39] reported that the in-plane large zigzag (LZZ) Na^+^/vacancy ordering in P2-type structures facilitates higher Na^+^ mobility, where a small fraction of Na^+^ migrates synergistically in a correlated manner, resulting in a decrease in energy barrier. They also suggested that the dynamic competition between ordering and disordering among corresponding ions can promote rapid ion diffusion, ultimately enhancing the high rate performance of rechargeable batteries. However, it is widely accepted that the Na^+^/vacancy ordering can also induce kinetic hysteresis, which presents a significant challenge to the electrochemical performance of P2-type layered oxides [40].

In this context, we propose and implement a novel regulation strategy for P2-Na_0.67_Ni_0.33_Mn_0.67_O_2_ by employing Cu/Y dual-site doping. This approach yields a promising new cathode material Na_0.67_Y_0.05_Ni_0.18_Cu_0.1_Mn_0.67_O_2_ (denoted as NYNCMO). In this design, Y is introduced into the AM layer, while Cu is substituted into the TM layer. Y^3+^ ions (0.9 Å) successfully enter the AM layer possibly due to their similar ionic radius to Na^+^ (1.02 Å), exerting a pillar effect that enhances the stability of structure. X-ray absorption spectroscopy (XAS) confirms that the oxidation state of Mn remains stable during charge/discharge cycles, effectively mitigating the structural degradation typically associated with the Jahn–Teller distortion. We further propose the formation of “Na–Y” aggregates as a novel type of interlayer pillar that reinforces structural integrity. Density functional theory (DFT) simulations support this concept, revealing a shortened Na–O bond length around Y and an increase in binding strength. This interaction prevents some Na^+^ in the original structure around Y from fully detaching during cycling, resulting in the formation of a stable interlayer pillar at the cost of a slight capacity trade-off.

Importantly, the presence of this novel interlayer pillar not only improves structural robustness but also helps in maintaining favorable Na⁺ diffusion kinetics, especially compared to traditional high sodium-content layered oxides. The excellent diffusion kinetics of Na^+^ are further validated by galvanostatic intermittent titration techniques (GITT) measurements and mean squared displacement (MSD) calculations. Another critical contribution of the interlayer pillar is its disruption of the ordered Na⁺/vacancy arrangement, inducing a mixed ordering–disordering state. This structural coexistence promotes faster Na⁺ diffusion and contributes to the outstanding rate performance. The optimized NYNCMO material delivers exceptional electrochemical results, achieving capacity retention rates of 71.7% and 76% at ultra-high rates of 20 C over 1600 cycles and 50 C over 1000 cycles, respectively. Additionally, it sustains 3000 (dis)charge cycles at 10 C within a voltage range of 2.0–4.0 V, demonstrating both remarkable rate capability and long-term cycling stability.

## Experiment Section

### Materials

Sodium acetate anhydrous (CH_3_COONa) was purchased from Sinopharm Chemical Reagent Co., Ltd. Nickel acetate tetrahydrate (Ni(CH_3_COO)_2_·4H_2_O) was purchased from Solarbio Science and Technology Co., Ltd. Manganese acetate tetrahydrate (Mn(CH_3_COO)_2_·4H_2_O) was purchased from Aladdin Industrial Corporation. Copper acetate monohydrate (Cu(CH_3_COO)_2_·H_2_O) was purchased from Shanghai Yuanye Bio-Technology Co., Ltd. Yttrium acetate hydrate Y(CH_3_COO)_3_·xH_2_O was purchased from Shanghai Yien Chemical Technology Co., Ltd. The anhydrous citric acid (C_6_H_8_O_7_) was purchased from Macklin Biochemical Co., Ltd. All of the chemicals were used directly without further purification.

### Sample Preparation

NYNCMO was synthesized using a straightforward sol–gel method. Stoichiometric amounts of CH_3_COONa (5 mol% excess), Cu(CH_3_COO)_2_·H_2_O, Y(CH_3_COO)_3_·xH_2_O, Ni(CH_3_COO)_2_·4H_2_O, Mn(CH_3_COO)_2_·4H_2_O were dissolved in a beaker with deionized water. Citric acid was dissolved in another beaker with the same volume of deionized water and controlled its dose to be 1.5 times that of the transition metal atoms. Then, the citric acid solution was pumped to the transition metal ions-contained solution drop by drop with continuous stirring until transparent. The entire process was carried out in a water bath environment at 80 °C and continuously stirred. The obtained gel was dried at 120 ℃ in the baking oven. Ultimately, the resulting gel was dried at 120 °C in an oven and then pre-calcined at 450 °C for 6 h in air using a muffle furnace. This was followed by a high-temperature calcination step at 900 °C for 15 h. The final product was immediately transferred to a glove box for storage. The comparative samples Na_0.67_Ni_0.23_Cu_0.1_Mn_0.67_O (denoted as NNCMO), Na_0.67_Y_0.05_Ni_0.28_Mn_0.67_O (denoted as NYNMO), Na_0.67_Ni_0.18_Cu_0.15_Mn_0.67_O (denoted as NNC_0.15_MO), and Na_0.67_Ni_0.33_Mn_0.67_O (denoted as NNMO) are synthesized using the same method as mentioned above.

### Characterization

The morphology of the samples was examined by scanning electron microscopy (SEM, Zeiss Gemini 300) and transmission electron microscopy (TEM, JOEL, JEM-2100F). The crystal structure of the as-obtained samples was analyzed using the powder X-ray diffractor (XRD, Ultima IV). X-ray photoelectron spectroscopy (XPS, ESCALAB 250XI) was used to determine the valence states of the elements.

In situ XRD measurements were performed using a custom-built cell with a beryllium window (Be window cell, Beijing SCISTAR Technology Co., Ltd.) to monitor structural evolution during cycling. Raman spectra were acquired with an inVia™ confocal Raman microscope (Renishaw) using a 633-nm argon ion laser. Ni K-edge and Mn K-edge X-ray absorption fine structure (XAFS) measurements were conducted at beamline BL14W1 of the Shanghai Synchrotron Radiation Facility (SSRF), using Si(111) crystal monochromators. Prior to analysis, samples were pressed into 1 cm-diameter pellets and sealed with Kapton tape. The spectra were collected at room temperature using a 4-channel Silicon Drift Detector (SDD, Bruker 5040) in transmission mode. Standard reference samples (NiO, Ni₂O₃, Mn₂O₃) were also analyzed. The XAFS data were processed and analyzed using the Athena software package.

### Electrochemical Measurements

Cathodes were fabricated by mixing 70% active material, 20% carbon black (Super-P), 10% polymer binder (polyvinylidene fluoride; PVDF), and N-Methyl pyrrolidone (NMP) together to form a slurry onto the aluminum foils and dried at 100 °C overnight in an air blast drying oven. Then, the electrode was cut into a specific size (12 mm in diameter). The electrode was cut into a specific size (12 mm in diameter). Coin cells (2032) were assembled in an Ar-filled MIKROUNA glovebox (O_2_ and H_2_O < 0.1 ppm), with sodium metal (diameter of 1.56 cm, thickness of ~450 μm) as reference electrode. The electrolyte was 1.0 M NaClO_4_ in propylene carbonate (PC) with 5 vol% of fluoroethylene carbonate (FEC), and Whatman glass microfiber filter (Grade GF/F) was used as the separator. Each cell used 120 μL of electrolyte.

Electrochemical performance was tested using the Land CT2001A battery test system (Wuhan, China) in the voltage range of 2.0–4.0 and 2.0–4.3 V. The cyclic voltammetry (CV) and electrochemical impedance spectroscopy (EIS) were performed by using a CHI660E electrochemical station under a potential region of 2.0–4.3 V and a frequency region of 100 kHz–10 mHz, respectively. Galvanostatic intermittent titration technology (GITT) tests were performed using a NEWARE test system with a 0.1 C charge/discharge current for 0.5 h. If the *Eτ* as a function of *τ* is linear, the Na^+^ diffusion coefficient can then be calculated by simplified Eq. (1):

1 $${D}_{{\mathrm{Na}}^{+}}=\frac{4}{\pi \tau }{\left(\frac{{m}_{B}{V}_{M}}{{M}_{B}A}\right)}^{2}{\left(\frac{{\Delta E}_{S}}{\Delta {E}_{\tau }}\right)}^{2}$$

where ${D}_{{\mathrm{Na}}^{+}}$ (cm^2^ s^−1^) means the chemical diffusion coefficient, *V*_*M*_ (cm^3^ mol^−1^); the molar volume, weight, and molar weight of the active materials are indicated by *m*_*B*_, *M*_*B*_, and *V*_*M*_, respectively. *A* and *τ* (*s*) represent the surface area of the electrode and the testing time in each step, and *ΔE*_*s*_, *ΔE*_*τ*_ are the quasiequilibrium potential and the change of cell voltage *E* during the current pulse, respectively.

### Computational Details

All calculations were performed by using the DFT method, as implemented in the Vienna Ab initio Simulation Package (VASP). The projector augmented-wave (PAW) method was used to describe ion–electron interactions. To accurately model the d orbitals of transition metals, the GGA + *U* approach was employed with effective *U* values of 6.1 eV for Ni, 3.8 eV for Mn, and 4.0 eV for Cu. The energy cut-off for the plane-wave basis set was 450 eV, and the energy and force convergence criteria for the relaxation were 10^–5^ eV per atom and 0.05 eV Å^−1^. Ab initio molecular dynamics (AIMD) simulations were carried out to evaluate Na⁺ diffusion in NNMO, NYNMO, NNCMO, and NYNCMO. The AIMD simulations are carried out for 7 ps at 700 K by a Nose–Hoover thermostat, and a time step of 1 fs was used, where the only Gamma point was used to keep the computational cost affordable. The diffusion probability of Na ions is realized by the pymatgen-analysis-diffusion code. The volume and the shape of the cell were fixed.

## Results and Discussion

### Structural and Morphological Characterization

The optimal Y content was determined through gradient experiments in the pre-experimental stage, as shown in Fig. S1.The designed P2-type NYNCMO electrode was synthesized using a simple and quick sol–gel method. Stoichiometric ratios of metal acetates were dissolved in deionized water, followed by the addition of citric acid as a chelating agent. After solvent evaporation, the precursor underwent high-temperature calcination to yield the final product. Details of the synthesis process are provided in Experimental Section. For comparison, a series of reference materials, including NNCMO, NYNMO, NNC_0.15_MO, and NNMO, were synthesized using the same method as NYNCMO. Their exact composition was examined by the inductively coupled plasma (ICP) method, as shown in Table S1, and the results have good consistency with the design system.

**Fig. 1:**
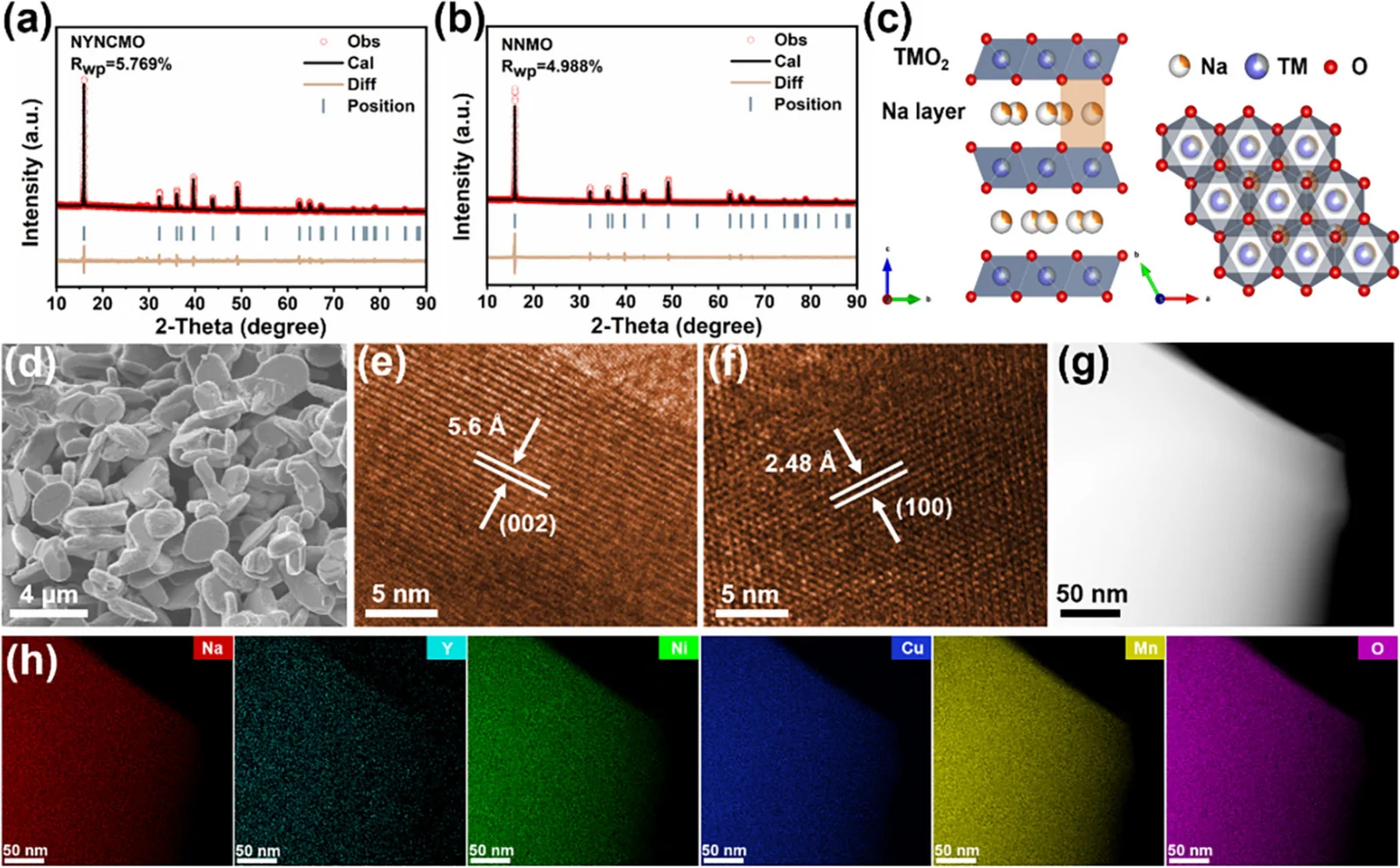
Structure characterization of P2-NYNCMO. XRD and Rietveld plots of **a** P2-NYNCMO and **b** P2-NNMO. **c** P2-type crystal structure viewed along the *b*-axis (left) and c-axis (right). **d** SEM image. **e, f** HRTEM analysis. **g** TEM image and **h** EDS mapping images of the P2-NYNCMO sample

X-ray diffraction (XRD) analysis reveals a notable shift of the (002) peak toward higher angles in the NYNCMO sample, indicating a contraction of the interlayer d-spacing along the *c*-axis (Fig. S2). To gain deeper insight into the structural characteristics, the Rietveld refinement was conducted on these samples. As shown in Figs. 1a, b and S3, the diffraction patterns are well indexed to a hexagonal structure (JCPDS-54-0894). Additionally, NYNCMO displays additional peaks between 25° and 30°, which can be attributed to the Na^+^ LZZ structure, as further discussed in Raman section. A schematic representation of the NYNCMO crystal structure (Fig. 1c) illustrates alternating TMO_2_ sheets and Na layers with an ABBA oxygen stacking sequence. When comparing the dual-doped sample (NYNCMO) with both singly doped Cu and pristine counterparts, the lattice parameters of NYNCMO (*a* = 2.8933 Å and *c* = 11.1586 Å) exhibit an expanded *a*–*b* plane and a contracted c-axis compared to NNCMO (*a* = 2.8867 Å, *c* = 11.1907 Å) and NNMO (*a* = 2.8713 Å, *c* = 11.1090 Å) (Tables S2–S7). This can be attributed to variations in the electrostatic cohesion between O^2−^ and O^2−^ in the NaO_2_ layer, suggesting the successful incorporation of dopant elements into the lattice, particularly into the NaO_2_ layer.

To determine the specific dopant site occupancy, Rietveld refinement was performed on two structures, as shown in Fig. S3d. The refinement results showed a marked increase in agreement factors when Cu was assumed as the element entering the AM layer. Therefore, we believe that it is the Y element that preferentially enters the AM layer. To further validate this finding, a compositionally tuned sample (NNC_0.15_MO) was synthesized to investigate the impact of varying Cu concentrations. Structural analysis of NNMO, NNCMO, and NNC_0.15_MO reveals that increasing Cu content leads to a gradual expansion of the c-axis. Since Cu incorporation into the AM layer would typically shield interlayer electrostatic repulsion and result in c-axis contraction, the observed expansion supports the conclusion that Cu occupies the transition metal layer. This finding aligns well with previous results, confirming the distinct roles and positions of Cu and Y dopants in the NYNCMO structure.

Scanning electron microscopy (SEM) images reveal that all the samples exhibit a plate-like particle morphology (Figs. 1d and S4). High-resolution transmission electron microscopy (HRTEM) analysis of the NYNCMO sample displays clear lattice fringes with interplanar spacings of 5.6 and 2.48 Å, corresponding to the (002) and (100) planes of the typical P2-type layered structure, respectively (Fig. 1e, f). The selected area electron diffraction (SAED) pattern further confirms the hexagonal layered structure, showing bright and regular diffraction spots along the [00 $\stackrel{\mathrm{-}}{1}$] axis (Fig. S5), which is consistent with the P2-type crystallography. Additionally, transmission electron microscopy (TEM) combined with energy dispersive spectrometry (EDS) elemental mapping of NYNCMO (Fig. 1g, h) confirms the presence and homogeneous distribution of Na, Y, Ni, Cu, Mn, and O throughout the plate-like particles without any aggregation, indicating successful incorporation of dopants and uniform phase formation.

**Fig. 2:**
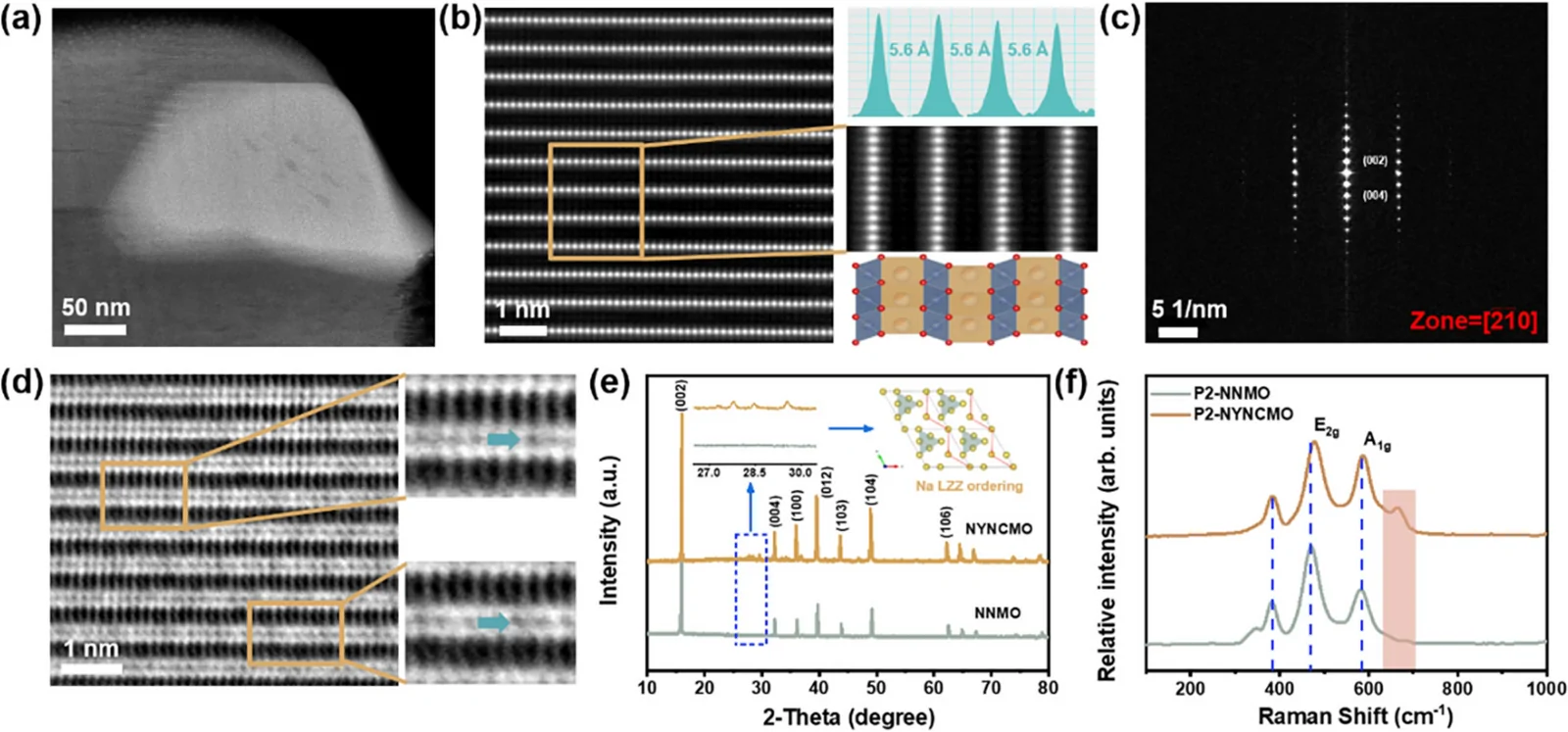
Spherical aberration corrected transmission electron microscope analysis and in-depth structural analysis of NYNCMO. **a** Selected TEM image for analysis. **b** HAADF–STEM image and corresponding structure diagram. **c** Corresponding FFT pattern collected from [$\stackrel{\mathrm{-}}{2}\stackrel{\mathrm{-}}{1}\mathrm{0]}$ axis. **d** ABF–STEM image and enlarged views of STEM images selected from specific areas (right). **e** XRD patterns of the as-prepared P2-NYNCMO and in-plane Na^+^/ vacancy ordering (internal illustration). **f** Raman spectra of P2-NYNCMO and P2-NNMO electrodes

To provide direct evidence of Y incorporation into the AM layer, ion thinning and spherical aberration-corrected transmission electron microscopy were performed on the edge of an individual NYNCMO particle (Fig. 2a). The high-angle annular dark-field scanning transmission electron microscopy (HAADF–STEM) image (Fig. 2b) reveals an interlayer spacing of approximately 5.6 Å between the TMO_2_ layers, consistent with HRTEM results and the structural configuration of P2-type oxides. This image is taken along the [$\stackrel{\mathrm{-}}{2}\stackrel{\mathrm{-}}{1}\mathrm{0]}$ crystal axis, and the corresponding fast Fourier transform (FFT) pattern is shown in Fig. 2c. A schematic of the crystal structure viewed along this axis is also provided in Fig. S6. The identification of the (002) and (004) planes is confirmed through a combination of inverse Fourier transform analysis and crystallographic symmetry considerations. Remarkably, two magnified annular bright-field STEM (ABF-STEM) images (Fig. 2d) show clear dark contrast features between neighboring TMO₂ layers. These dark patches are compelling evidence of Y occupancy in the Na layer, confirming the formation of interlayer pillar structures. Previous studies have reported the presence of Na^+^ LZZ ordering in P2-type layered oxides [41, 42]. According to Shi's et al. [39], the disruption of Na⁺/vacancy ordering eliminates characteristic superstructure peaks in the XRD pattern between 25° and 30°. In contrast, NYNCMO retains these superstructure peaks (Fig. 2e), indicating the persistence of Na^+^ LZZ ordering in the material.

To further explore the Na⁺ spatial arrangement, Raman spectroscopy was conducted on the NYNCMO, and the resulting Raman spectra are presented in Fig. 2f. For the P63/mmc space group, the Raman active optical modes are given by *Γ*(Raman, optic) = A_1g_ + 3E_2g_ + E_1g_. The E_2g_ modes are associated with Na⁺ vibrations at 2*b* and 2*d* sites, whereas the A_1g_ and E_1g_ modes correspond to oxygen vibrations at 4*f* sites. According to polarized Raman studies by Qu et al. [43], the E_2g_ mode appears near 460 cm^−1^ and the A_1g_ mode near 576 cm^−1^ in Na_x_CoO_2_. In NYNCMO, these peaks are slightly shifted to 470 cm^−1^ (E_2g_) and 582 cm^−1^ (A_1g_), indicating a preserved but modified lattice environment. Notably, an additional broad Raman peak is observed at 665 cm^−1^, which has also been reported in disordered P2-Na_2/3_Al_1/24_Ni_7/24_Mn_2/3_O_2_ structures [44]. This peak is attributed to the emergence of disordered Na^+^ arrangements and possible sublattice formation. We interpret this as indirect evidence for a partial transformation from an ordered Na^+^/vacancy configuration to a disordered state. Therefore, we conclude that NYNCMO exhibits a unique Na⁺ configuration characterized by a coexistence of ordered and disordered Na⁺/vacancy domains, an arrangement that likely contributes to its enhanced Na⁺ diffusion kinetics and electrochemical performance.

### Electrochemical Performance

**Fig. 3:**
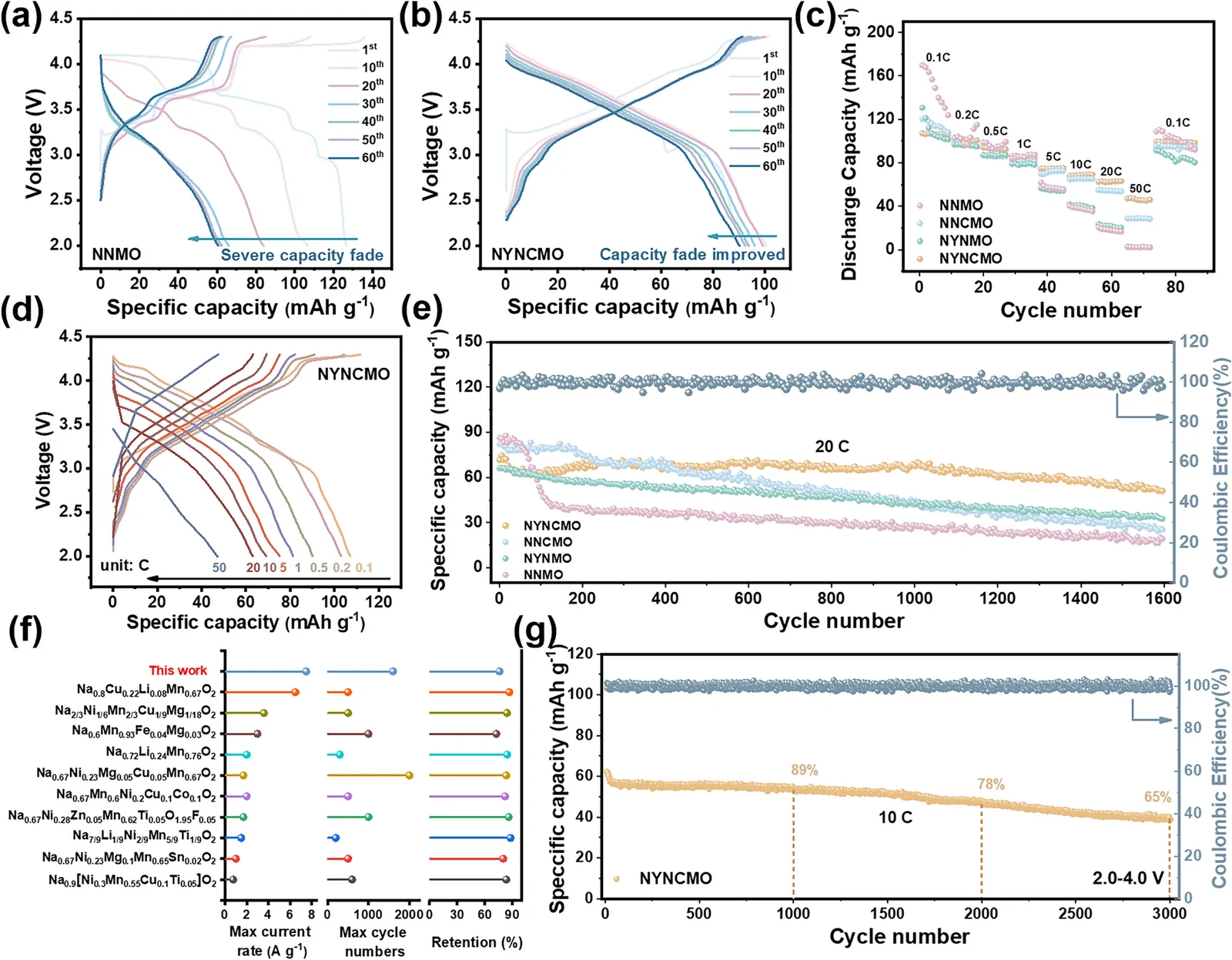
Electrochemical performance of the cathode materials. Charge/discharge curves of **a** NNMO and **b** NYNCMO at 1 C during 60 cycles. **c** Rate performance of NNMO, NNCMO, NYNMO, and NYNCMO cathodes at 0.1, 0.2, 0.5, 1, 5, 10, 20, and 50 C, respectively. **d** Charge/discharge curves of NYNCMO at corresponding rates. **e** Long-term cycle performance comparison of NNMO, NNCMO, NYNMO, and NYNCMO cathodes at an ultra-high rate of 20 C. **f** Comparison of electrochemical properties between NYNCMO and other reported P2-type oxide cathodes. **g** Electrochemical performance of NYNCMO at 10 C in the voltage range of 2.0–4.0 V

The electrochemical properties of the NYNCMO electrode in half cells were evaluated over a wide voltage range of 2.0–4.3 V to investigate the effects of Cu/Y dual-site doping. Figure 3a, b presents the charge/discharge profiles of NNMO and NYNCMO electrodes over the first 60 cycles at a current density of 1 C (1 C = 150 mAh g^−1^). The P2-NNMO electrode exhibits multiple voltage plateaus, particularly below 4.0 V, which are typically attributed to Na^+^/vacancy ordering. This ordering hinders Na^+^ diffusion, resulting in poor rate performance. In contrast, the plateau above 4.0 V is associated with the P2–O2 phase transition, known to induce irreversible structural degradation and rapid capacity fading [12, 45].

Compared to NNMO, the P2-NYNCMO electrode shows a smoother voltage profile and a significantly shorter high-voltage plateau, indicating that the challenges of Na^+^/vacancy ordering and the adverse P2–O2 phase transition are effectively suppressed through Cu/Y doping. Enhanced capacity retention and more stable cycling behavior were observed, consistent with the cyclic voltammetry (CV) results (Fig. S7). The coexistence of ordered and disordered Na⁺/vacancy arrangements in NYNCMO is also reflected in its charge/discharge curves. Meng et al. [41] identified two ordered structures in P2-Na_x_Ni_1/3_Mn_2/3_O_2_ at around 3.5 and 4.0 V, both visible in the NNMO profile. However, we observe that the ~3.5 V plateau disappears in NYNCMO, indicating a transition from ordered to disordered Na^+^/vacancy arrangements due to Y substitution. However, the ~4.0 V feature remains, suggesting a partial ordering persists. This supports the structural findings from XRD and Raman analyses, highlighting a mixed ordering–disordering state, which facilitates faster Na⁺ kinetics and enhances rate capability.

The rate behaviors of NNMO, NYNMO, NNCMO, and NYNCMO materials are shown in Fig. 3c. The corresponding charge/discharge curves for P2-NYNCMO and P2-NNMO at different rates are provided in Figs. 3d and S8. The P2-NYNCMO electrode demonstrates the best rate performance among the involved materials. Specifically, it delivers reversible discharge capacities of 111.6, 104.4, 91, 86.1, 74.7, 72.5, 71.7, and 53.3 mAh g^−1^ at 0.1, 0.2, 0.5, 1, 2, 5, 10, 20, and 50 C, respectively. In contrast, the P2-NNMO electrode exhibits much poorer performance, retaining only 20.4 and 2.5 mAh g^−1^ at 20 C and 50 C, respectively. This dramatic enhancement in rate performance is attributed to its solid solution reaction mechanism and improved Na⁺ diffusion kinetics [38], which will be further discussed in subsequent sections. Cycling stability tests for P2-NNMO and P2-NYNCMO at 1 C and 5 C are presented in Fig. S9. The P2-NYNCMO electrode maintains 89% and 85.3% of its initial capacity after 100 and 500 cycles, respectively, significantly outperforming the undoped NNMO. Moreover, long-term cycling at 20 C (Fig. 3e) demonstrated that NYNCMO retains 71.7% of its initial capacity after 1600 cycles, whereas the other three compositions experience rapid capacity fading.

Under ultra-high-rate conditions (50 C), the NYNCMO electrode delivers an initial capacity of ~70 mAh g^−1^ and maintains ~52 mAh g^−1^ after 1000 cycles, corresponding to a capacity retention of ~76% (Fig. S10). These results confirm its excellent long-term cycling stability and high-rate capability. In comparison with previously reported P2-type oxide cathodes, the Cu/Y dual-site doped NYNCMO exhibits superior electrochemical performance (Fig. 3f, Table S8) [20, 27, 46–53]. Remarkably, NYNCMO achieves ultra-long cycling stability, sustaining 65% capacity retention after 3000 cycles at 10 C (Fig. 3g). The average capacity decay is only 0.012% per cycle, showing excellent structural stability.

### Structural Evolution and Reaction Mechanism

**Fig. 4:**
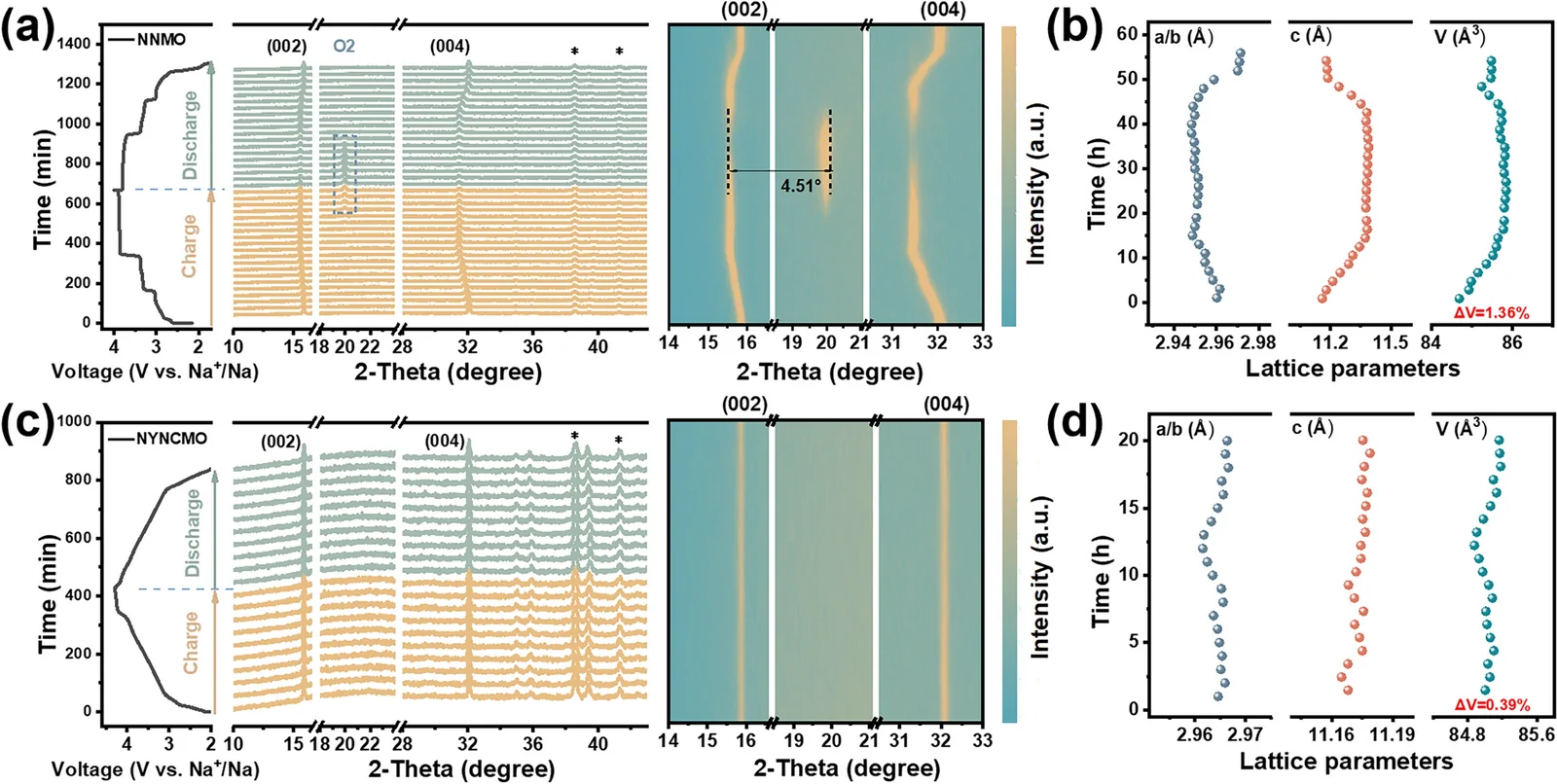
Structural evolution and analysis. **a** In situ XRD patterns collected during the first charge/discharge of the P2-NNMO electrode and the corresponding intensity contour map. **b** Variation in lattice parameters *a, c,* and *V* of NNMO. **c** In-situ XRD patterns collected during the first charge/discharge of the P2-NYNCMO electrode and the corresponding intensity contour map. **d** Variation in lattice parameters a, c, and V of NYNCMO

To investigate the structural evolution of the materials during electrochemical cycling, in situ XRD was conducted for both NNMO and NYNCMO electrodes during the first charge/discharge cycle within the voltage range of 2.0 and 4.3 V (Fig. 4). For NNMO, the (002) and (004) diffraction peaks constantly shift toward lower angles upon charging (Fig. 4a), indicating an expansion along the *c*-axis due to increased electrostatic repulsion between adjacent oxygen layers as Na⁺ ions are extracted. These peaks return to their original positions during the discharge, reflecting reversible lattice breathing behavior. However, when charged above 4.2 V, a new prominent peak appears around 20°, clearly evidencing a severe P2–O2 phase transition. This irreversible structural damage caused by severe phase transition results in poor electrochemical performance. In contrast, the NYNCMO electrode exhibits only slight shifts in the (002) and (004) peaks during the whole cycling, with no emergence of new peaks or significant peak shape changes (Fig. 4c), indicating the absence of phase transitions and a stable solid solution-type reaction mechanism. This observation aligns with the smooth, sloped electrochemical profiles observed for NYNCMO, consistent with single-phase behavior.

To quantify the structural changes, we monitored the evolution of lattice parameters (*a* and *c*) during the charging/discharging process (Fig. 4b, d). For NYNCMO, both lattice parameters (*a* and *c*) and unit cell volume change linearly with the charge/discharge voltage, indicating the sustainable evolution and single-phase reaction. Notably, the overall lattice volume change is ~0.39%, highlighting the near-zero strain characteristics of NYNCMO during Na^+^ insertion and extraction. The excellent structural stability also enables it to exhibit outstanding cycling stability even at high current densities. We attribute this remarkable structural stability to the presence of aggregates composed of Y and Na^+^ in the AM layer. As shown by the following calculations, the Na–O bond energy around Y is enhanced. The system containing this aggregate exhibits lower total energy and is more likely to remain stable, demonstrating its effectiveness in suppressing the P2–O2 phase transition from an energetic perspective.

To further evaluate the impact of phase transitions on the structural integrity, SEM was performed on NNMO, NNCMO, NYNMO, and NYNCMO samples after 100 cycles at 1 C (Fig. S11). The results reveal that only NYNCMO maintains a well-preserved layered structure, while the other samples exhibit pronounced microcracking and degradation. This observation provides direct evidence of NYNCMO’s superior structural robustness, attributed to the stabilizing effect of Y doping in the AM layer. The excellent structural stability of NYNCMO directly contributes to its outstanding long-term cycling performance, even under high current densities, affirming the effectiveness of the Cu/Y dual-site doping strategy in mitigating phase transitions and preserving electrode integrity.

### Na^+^ Kinetics and Reaction Mechanism Study

**Fig. 5:**
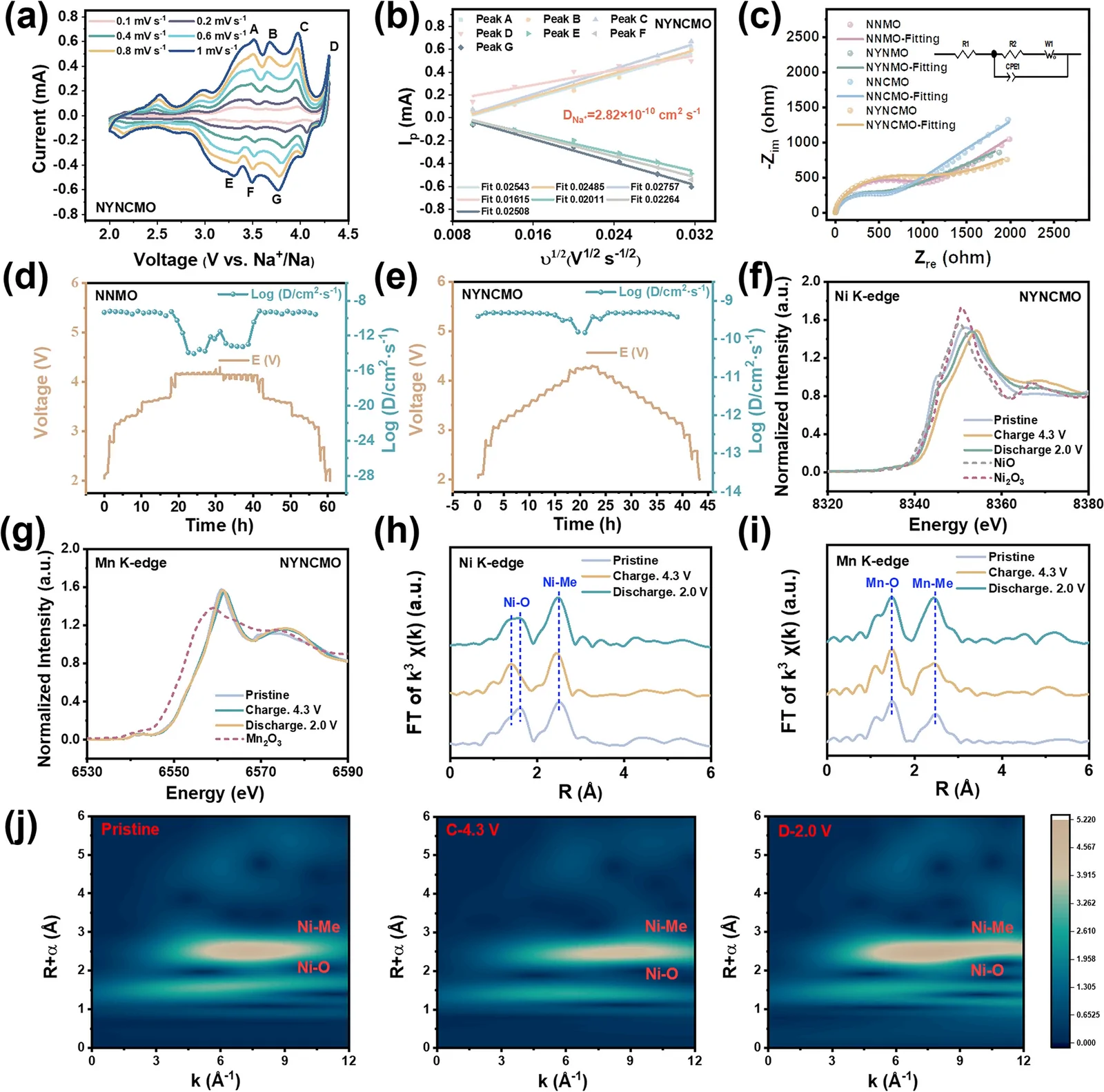
Na^+^ kinetics analyzes and charge compensation mechanism of P2-NYNCMO electrode. **a** Multisweep CVs of NYNCMO at various scan rates and **b** the corresponding cycling response of different peaks. **c** Nyquist plots of the NNMO, NNCMO, NYNMO, and NYNCMO cathodes (the internal illustration shows an equivalent circuit diagram). GITT curves of **d** NNMO and **e** NYNCMO electrodes. Ex-situ XANES spectra at the **f** Ni K-edge and **g** Mn K-edge of P2-NYNCMO electrode collected at different charge/ discharge states. Ex-situ EXAFS spectra at the **h** Ni K-edge and **i** Mn K-edge of P2-NYNCMO electrode collected at different charge/discharge states. FT = Fourier transform. **j** Wavelet transform (WT) contour plots of Ni in NYNCMO-pristine, charge to 4.3 V, and discharge to 2.0 V

To elucidate the electrochemical reaction mechanism, multisweep cyclic voltammetry (CV) measurements were performed on the four electrode materials (NNMO, NYNMO, NNCMO, and NYNCMO) within a voltage range of 2.0–4.3 V at scan rates ranging from 0.1 to 1 mV s^−1^ (Figs. 5a, S12). Among these, NYNCMO exhibits significantly higher current responses while maintaining a consistent curve shape across various scan rates, indicating low polarization and stable electrochemical kinetics. In contrast, NNMO, NYNMO, and NNCMO show smaller current responses and some redox peaks vanish at higher scan rates, suggesting the presence of irreversible electrochemical reactions, such as bulk phase transitions or surface degradation. The peak current ($I_{{\mathrm{P}}}$) of NYNCMO as a function of the square root of the scan rate ($\nu^{1/2}$) displays a linear relationship (Fig. 5b), which confirms that the apparent Na⁺ diffusion coefficient ${\mathrm{D}}_{{\mathrm{Na}}^{+}}$ remains relatively constant across different scan rates. Using the Randles–Sevcik equation [Eq. (2)], a stable ${\mathrm{D}}_{{\mathrm{Na}}^{+}}$ of 2.82 × 10^−10^ cm^2^ s^−1^ was obtained for NYNCMO.

2 $${I}_{\mathrm{P}}=0.4463nFAC{\left(\frac{NFvD}{RT}\right)}^\frac{1}{2}$$

Among all the involved electrodes, the high ${\mathrm{D}}_{{\mathrm{Na}}^{+}}$ value demonstrates the fast Na⁺ transport capability of NYNCMO.

To explore the Faradaic behavior, CV curves of all four cathodes were recorded over the first five cycles at a scan rate of 0.2 mV s^−1^ within the voltage range of 2.0–4.3 V (vs. Na^+^/Na) (Fig. S7). The NYNCMO electrode displays highly reproducible redox profiles with minimal peak fading, indicating stable and reversible electrochemical behavior. In contrast, NNMO, NNCMO, and NYNMO exhibit noticeable degradation in their reduction peaks, especially in the high-voltage region, which is likely due to some irreversible processes such as an irreversible bulk structure change or surface side reactions. Electrochemical impedance spectroscopy (EIS) was also performed to further investigate the Na^+^ diffusion kinetics (Fig. 5c). The fitted Nyquist plots, based on an equivalent circuit model, show that NYNCMO possesses the lowest series resistance (*R*_s_ = 4.001 Ω) and charge transfer resistance (*R*_ct_ = 222.8 Ω) among the four materials (Table S9). This indicates superior electron conductivity and faster interfacial charge transfer, further supporting the enhanced electrochemical performance of NYNCMO.

To quantitatively analyze Na⁺ diffusion, galvanostatic intermittent titration technique (GITT) measurements were carried out (Fig. 5d, e). The GITT profile of P2-NNMO shows a notable voltage plateau, particularly at the high voltages, indicating a severe P2–O2 phase transition. In contrast, P2-NYNCMO exhibits a continuous change in equilibrium potential, reflecting a solid-solution reaction mechanism and stable electrochemical kinetics throughout the charge/discharge process. From the GITT data, the ${\mathrm{D}}_{{\mathrm{Na}}^{+}}$ of ~4.25 × 10^–10^ cm^2^ s^−1^ is obtained for P2 NYNCMO, which is an order of magnitude higher than that of P2-NNMO (3.64 × 10^–11^ cm^2^ s^−1^). The rapid diffusion kinetics of Na^+^ can also decrease material polarization and alleviate local environmental stress, which indirectly helps maintain the integrity of the structure. In summary, NYNCMO demonstrates significantly lower charge transfer resistance, higher Na⁺ diffusion coefficients, and stable kinetic behavior, all of which are consistent with its superior electrochemical performance and our subsequent computational findings.

### Charge Compensation During Charge/Discharge

To further understand the charge compensation mechanism in the NYNCMO electrode during Na^+^ de/intercalation, ex situ X-ray absorption spectroscopy (XAS) and ex situ X-ray photoelectron spectroscopy (XPS) were conducted. The normalized X-ray absorption near edge structure (XANES) spectra at the Ni and Mn K-edges under different charging/discharging states are presented in Fig. 5f, g. As shown in Fig. 5f, the Ni K-edge absorption edge of NYNCMO lies close to that of NiO standard spectrum. Upon charging to 4.3 V, the absorption edge shifts to higher energy, indicating the progressive oxidation of Ni ions toward a +4 valence state. This shift is reversed during discharge, suggesting a highly reversible Ni redox process. A shoulder peak with an intensity of ~1.0 appears in both the pristine and discharged states but disappears in the charged state. This feature is attributed to the Jahn–Teller distortion effect of Ni^3+^. Upon oxidation during charging, this distortion vanishes as Ni transitions to a more symmetric configuration, leading to the disappearance of the shoulder. We believe that the failure to restore the pristine state in the K-edge spectrum of Ni at discharge state is due to the formation of peroxo-like oxygen (O_2_)^n−^ generated by the redox of oxygen at high voltage. These species locally stabilize the Ni–O framework, preventing full structural relaxation. Extended X-ray absorption fine structure (EXAFS) data results also provide evidence to support it [54]. In contrast, the Mn K-edge spectrum (Fig. 5g) remains unchanged throughout the electrochemical cycle, confirming the electrochemical inactivity of Mn^4+^ in P2-NYNCMO. To gain further insight into the local structural environment, EXAFS analysis was carried out at the Ni and Mn K-edges (Fig. 5h, i). In layered oxides, the two dominant EXAFS peaks are known to be indexed to TM–O in the first coordination shell and TM–TM in the second shell. For Ni, a decrease in interatomic distance and a reduction in Fourier transform (FT) amplitude in the first Ni–O coordination shell are observed, indicative of local structural distortion associated with charge compensation during the redox process. In contrast, only slight changes are observed at the Mn K-edge, again reinforcing that Mn remains structurally and electrochemically inert, still indicating that the redox active center is Ni. Upon discharged to 2.0 V, the Ni–O peak broadens and spans the peak widths of both the pristine state and charged states, showing a bimodal distribution. This demonstrate that some Ni–O bonds reversibly return to the pristine state, while other retain the characteristics of the charged state, leading to an irreversible shortening of Ni–O bonds. As a result, the K-edge absorption spectrum of Ni in the discharge state fails to fully revert to that of the pristine state. These XANES and EXAFS results are consistent with the in situ XRD results, where the variations in the lattice parameter correspond to Ni redox activity. To visualize changes in the covalent environment of TM-O and TM–TM more intuitively, the wavelet transform (WT) contour plots of Ni and Mn K-edge EXAFS were also generated (Figs. 5j, S13). The reduction in intensity and radial distance shift of Ni’s TM-O scattering peak further confirms valence state-induced changes in Ni’s coordination environment. Meanwhile, Mn shows only minor intensity changes, supporting its redox inactivity.

Ex situ XPS measurements were also performed to analyze surface chemical composition and valence states of the elements in NYNCMO. The Mn 2*p* spectrum (Fig. S14) shows two distinct peaks at 642.06 and 653.70 eV, corresponding to the Mn^4+^ valence state. These peak positions remain unchanged during the charge/discharge process, confirming that Mn maintains a +4 oxidation state, in contrast with previously reported coexistence of Mn^3+^/Mn^4+^ in NNMO. The absence of the Mn^3+^ signal means its rather low content, which suppresses the Jahn–Teller distortion, contributing significantly to the structural stability of the NYNCMO. XPS analysis of Ni (Fig. S15) reveals a reversible redox process following the Ni^2+^ → Ni^3+^ → Ni^4+^ pathway during electrochemical cycling, confirming that Ni is the primary center of the redox reaction and the source of charge compensation in the NYNCMO cathode material. Additionally, the O 1*s* spectra of NYNCMO materials in different states are also tested (Fig. S16). The peak at ~532.2 eV corresponds to lattice oxygen, which maintains good intensity across various states, indicating that the structural stability of the material has been improved compared to the reported NNMO. Notably, upon charging to 4.3 V, the peak around 533.7 eV can be attributed to peroxo-like oxygen (O_2_)^n−^, demonstrating that there is also a certain degree of oxygen redox reaction in the system, which makes an additional contribution to the capacity. The presence of peroxides also changes the environment of the surrounding metals. Moreover, the high-resolution Cu 2*p* spectrum (Fig. S17) shows two characteristic peaks at 933.18 and 952.92 eV, along with satellite features at 943.40 and 961.35 eV, indicating that Cu exists in a divalent (Cu2⁺) state. Cu plays an important role in optimizing the charge distribution and electronic structure within transition metal layers. By partially substituting Ni with Cu, the Mn^3+^ concentration was lowered to preserve the electrode material's overall charge balance so that the Jahn–Teller distortion effect of Mn is suppressed. Likewise, Y 3*d* spectra (Fig. S18) show two peaks at 156.1 and 158.1 eV, characteristic of the +3 valence state of Y, while a nearby peak at 153.2 eV can be attributed to an overlapping Si 2*s* interference peak. Importantly, the valence states of both Cu and Y remain unchanged during the charging–discharging process, suggesting that these dopants are electrochemically inactive. This further demonstrates that Ni is the redox-active species and the primary component responsible for charge compensation in NYNCMO. These findings are fully consistent with the results obtained from XAS.

### DFT Calculation and Simulations

**Fig. 6:**
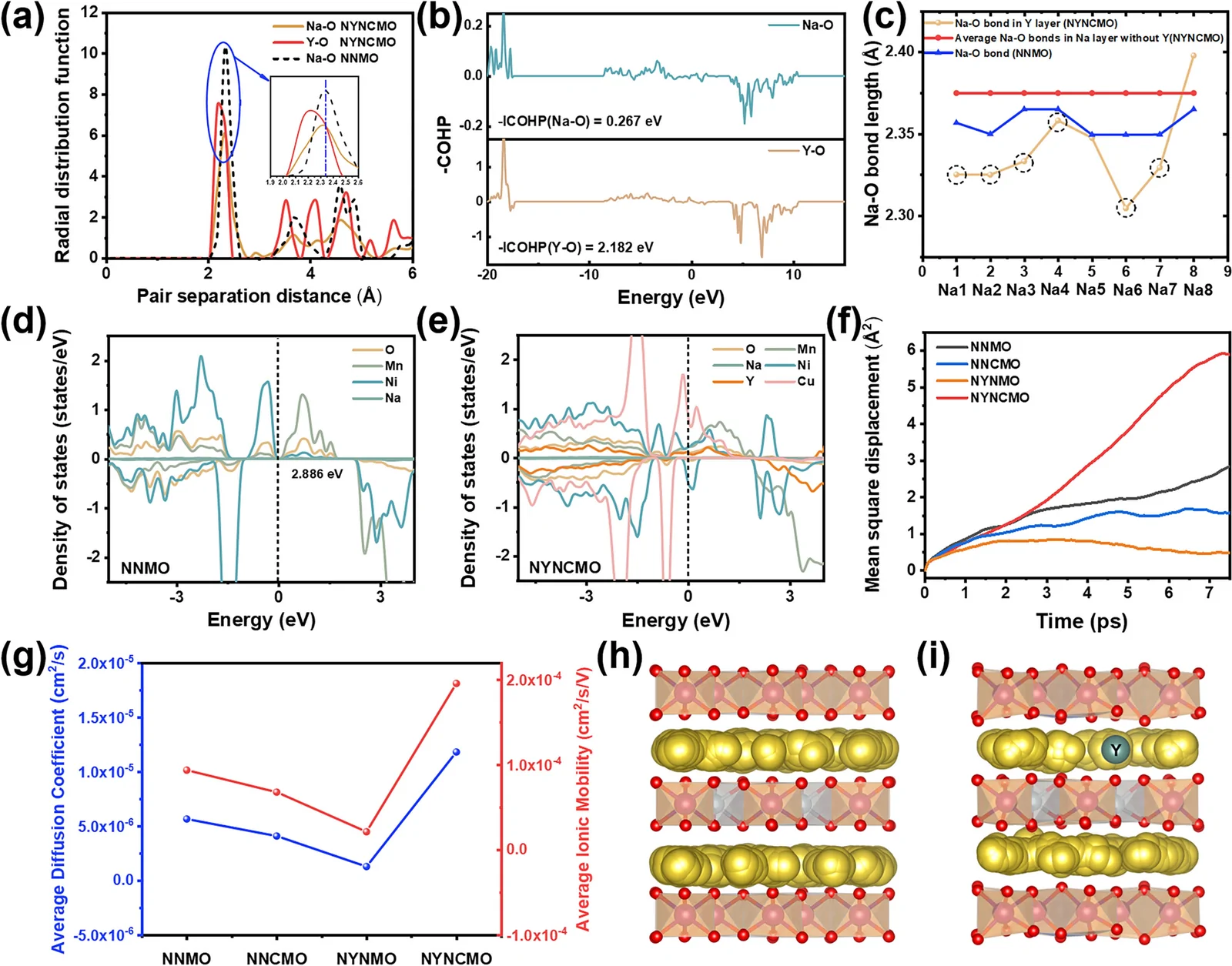
DFT calculation.** a** Radial distribution function of Y–O and Na–O in different structures. **b** COHP of Na–O and Y–O bonds in NYNCMO. **c** Comparison of Na–O bond length in different environments. Normalized partial density of states (pDOS) of **d** NNMO and **e** NYNCMO. **f** Comparison of total mean square displacement of Na ions in four materials. **g** Calculation results of the average Na^+^ diffusion coefficient and average ion mobility of the four materials. Trajectories of Na^+^ in **h** NNMO and **i** NYNCMO simulated at a temperature of 700 K over a period of 7 ps

To investigate the effect of selective Cu/Y dual-site doping on the AM layer, radial distribution function (RDF) analysis was performed to evaluate bond length distributions in NNMO and NYNCMO structures (Fig. 6a). The initial RDF peaks reveal that the Y–O bond length in NYNCMO is slightly shorter than the Na–O bond length, as indicated in the inset of Fig. 6a. This suggests a stronger local interaction between Y and oxygen atoms when Y is incorporated into the AM layer. To further probe this bonding behavior, the crystal orbital Hamilton populations (COHP) analysis was carried out. The integrated COHP (-ICOHP) values show that the Y–O bond possesses significantly greater bonding strength than the Na–O bond (Fig. 6b). Additionally, comparison of Na–O bond length distributions in NNMO and NYNCMO reveals that the position of Na–O bond is marginally smaller in NYNCMO (Fig. 6a), which suggests that Na–O bond lengths in NYNCMO are shorter, implying the presence of Y in the AM layer strengthens nearby Na–O bonds. This increased binding strength makes it more difficult for Na^+^ to be extracted from these positions, resulting in the formation of a tiny quantity of "Na–Y" aggregates, which act as novel interlayer pillars that reinforce the structural integrity of the electrode. To validate the above hypothesis, we analyzed 8 Na sites adjacent to Y and compared their Na–O bond lengths (Figs. 6c, S19). The majority of these Na–O bond lengths in NYNCMO are shorter than those in Y free regions and the NNMO structure, providing further evidence for the formation of "Na–Y" aggregates. These aggregates are also believed to contribute to the transition from an ordered to disordered Na⁺/vacancy arrangement, due to the localized immobilization of Na⁺ ions near Y sites. Moreover, the total energy of P2 and O2 phases with and without "Na–Y" aggregates is calculated (Fig. S20). The results show that the P2 phase is more thermodynamically stable (lower total energy) in the presence of "Na–Y" aggregates, whereas the O2 phase is more stable in their absence. This aligns with the observation that the P2–O2 phase transition is more likely to occur in NNMO. In addition, the comparison of phase stability can also be obtained through generalized stacking fault energy [55]. Overall, the presence of "Na–Y" aggregates contributes significantly to the thermodynamic stabilization of the P2 phase.

DFT calculations were used to investigate the impact of dual-site doping on electronic structures of the materials (Fig. 6d, e). NNMO exhibits half-metallic behavior, with the spin-up state being metallic (having no bandgap) and the spin-down state showing a bandgap of 2.886 eV. After dual-site doping, a new split energy level appears in the spin-down state's bandgap of NYNCMO, causing the density of states to shift toward a continuous distribution at the Fermi level. This shift indicates a substantial improvement in the electronic conductivity of NYNCMO, which enhances its electrochemical performance. To further examine Na⁺ transport behavior, MSD analysis was performed for all four materials: NNMO, NYNMO, NNCMO, and NYNCMO, based on AIMD simulations (Fig. S21). The lowest MSD values in the z-direction for all materials confirm that Na^+^ diffusion predominantly occurs in the two-dimensional plane (*a–b* plane), consistent with typical interlayer diffusion in rocking chair-type SIBs. Among all samples, NYNCMO exhibits the highest total MSD (Fig. 6f), indicating superior Na^+^ diffusion behavior.

The diffusion coefficients and ionic mobilities of Na⁺ for the four materials were also calculated (Tables S10, S11). NYNCMO exhibits the highest values for both parameters (Fig. 6g), confirming that Cu/Y dual-site doping significantly enhances Na^+^ diffusion kinetics. Additionally, AIMD simulations were performed to visualize the Na⁺ migration pathways in NNMO and NYNCMO (Fig. 6h, i). It demonstrates that Na^+^ migrates in the two-dimensional *a–b* plane. In NYNCMO, Na^+^ ions exhibit more interconnected diffusion trajectories across the *a–b* plane within the same simulation time, compared to NNMO. Interestingly, isolated "island-like" regions were observed in the Na^+^ diffusion maps around Y-containing AM layers (Fig. S22), suggesting that "Na–Y" aggregates locally hinder Na^+^ diffusion. However, in Y-free AM regions of NYNCMO, expanded interlayer spacing facilitates Na^+^ diffusion (Fig. S23), resulting in an overall enhancement in transport behavior. In conclusion, the formation of "Na–Y" aggregates introduces a significant pillar effect in the AM layer, which not only reinforces the structural stability of the layered oxide framework but also optimizes Na⁺ migration through a synergistic balance of diffusion restriction and pathway facilitation in the dual-site doped NYNCMO system.

## Conclusions

In summary, the designed Na_0.67_Y_0.05_Ni_0.18_Cu_0.1_Mn_0.67_O_2_ cathode, engineered through the regulation of alkali metal layers, demonstrates exceptional performance, including superior rate capability (achieving 70 mAh g^−1^ at 50 C) and ultra-stable cycling performance (sustaining 3000 cycles at 10 C). XAS confirms that the redox process is primarily governed by Ni, effectively avoiding the Jahn–Teller distortion typically induced by Mn. Importantly, in situ XRD, GITT, and molecular dynamics simulations collectively demonstrate that the novel "Na–Y" interlayer pillar successfully mitigates adverse phase transitions and enhances Na^+^ diffusion kinetics within the structure. Further supporting this, the XRD characterization, Raman spectroscopy, simulation of diffusion trajectories, and the disappearance of the characteristic Na^+^/vacancy ordering plateau in the charge/discharge curve provide direct evidence for the coexistence of ordered and disordered Na^+^/vacancy states. The coexistence of Na^+^/vacancy ordering–disordering states, facilitated by this novel interlayer pillar, significantly enhances Na^+^ diffusion kinetics and provides valuable insights into the dynamics of Na^+^/vacancy interactions. We believe that this discovery of a unique interlayer pillar will open new avenues for the regulation engineering of AM layers in sodium-ion battery cathodes. It holds the potential to inspire the design of various interlayer pillar combinations aimed at further improving the structural stability of layered oxide cathode materials. Overall, these findings offer fresh perspectives and effective strategies for the development of high-rate, long-life electrode materials for next-generation sodium-ion batteries.

## Supplementary Information

Below is the link to the electronic supplementary material.

Supplementary file1 (DOCX 8763 kb)

## References

[1] B. Dunn, H. Kamath, J.-M. Tarascon, Electrical energy storage for the grid: a battery of choices. Science **334**(6058), 928–935 (2011). 10.1126/science.1212741 22096188 10.1126/science.1212741

[2] Z. Zhao, Y. Wu, R. Hu, J. Lu, D. Chen et al., Intercalation pseudocapacitance in 2D VS_2_/Ti_3_C_2_T_*x*_ MXene hybrids for all-climate and long-cycle sodium-ion batteries. Adv. Funct. Mater. **33**(50), 2307794 (2023). 10.1002/adfm.202307794

[3] X. Rong, J. Liu, E. Hu, Y. Liu, Y. Wang et al., Structure-induced reversible anionic redox activity in Na layered oxide cathode. Joule **2**(1), 125 (2018). 10.1016/j.joule.2017.10.008

[4] D. Chen, Y. Xu, J. Lu, Y. Tian, T. Li et al., Intercalation-induced localized conversion reaction in h-CuSe for ultrafast-rechargeable and long-cycling sodium metal battery. Adv. Mater. **36**(32), 2404640 (2024). 10.1002/adma.202404640 10.1002/adma.202404640 38775475

[5] H.Y. Asl, A. Manthiram, Reining in dissolved transition-metal ions. Science **369**(6500), 140–141 (2020). 10.1126/science.abc5454 32646985 10.1126/science.abc5454

[6] K. Kubota, S. Komaba, Review: practical issues and future perspective for Na-ion batteries. J. Electrochem. Soc. **162**(14), A2538–A2550 (2015). 10.1149/2.0151514jes

[7] Z. Yang, Y. Lu, H. Dong, J. Lin, Y. Wang, M. Qiu, Z. Ye, J. Lu, Recent progress and prospect of Li-Se batteries: a comprehensive review. Energy Mater. **3**, 300027 (2023). 10.20517/energymater.2022.91

[8] H. Kim, H. Kim, Z. Ding, M.H. Lee, K. Lim et al., Recent progress in electrode materials for sodium-ion batteries. Adv. Energy Mater. **6**(19), 1600943 (2016). 10.1002/aenm.201600943

[9] M.H. Han, E. Gonzalo, G. Singh, T. Rojo, A comprehensive review of sodium layered oxides: powerful cathodes for Na-ion batteries. Energy Environ. Sci. **8**(1), 81–102 (2015). 10.1039/C4EE03192J

[10] X. Wang, L. Ren, Y. Wang, M. Qiu, Z. Yang et al., A strategy to enhance rate capability by doping Titanium into Na_2_FeP_2_O_7_@C cathode materials for Na-ion batteries. J. Power. Sources **557**, 232533 (2023). 10.1016/j.jpowsour.2022.232533

[11] K. Kubota, S. Kumakura, Y. Yoda, K. Kuroki, S. Komaba, Electrochemistry and solid-state chemistry of NaMeO_2_ (me = 3d transition metals). Adv. Energy Mater. **8**(17), 1703415 (2018). 10.1002/aenm.201703415

[12] Q. Liu, Z. Hu, M. Chen, C. Zou, H. Jin et al., P2-type Na_2/3_Ni_1/3_Mn_2/3_O_2_ as a cathode material with high-rate and long-life for sodium ion storage. J. Mater. Chem. A **7**(15), 9215–9221 (2019). 10.1039/C8TA11927A

[13] S. Komaba, T. Nakayama, A. Ogata, T. Shimizu, C. Takei et al., Electrochemically reversible sodium intercalation of layered NaNi_0.5_Mn_0.5_O_2_ and NaCrO_2_. ECS Trans. **16**(42), 43–55 (2009). 10.1149/1.3112727

[14] N. Ortiz-Vitoriano, N.E. Drewett, E. Gonzalo, T. Rojo, High performance manganese-based layered oxide cathodes: overcoming the challenges of sodium ion batteries. Energy Environ. Sci. **10**(5), 1051–1074 (2017). 10.1039/C7EE00566K

[15] P.-F. Wang, Y. You, Y.-X. Yin, Y.-G. Guo, Layered oxide cathodes for sodium-ion batteries: phase transition, air stability, and performance. Adv. Energy Mater. **8**(8), 1701912 (2018). 10.1002/aenm.201701912

[16] X. Wang, Z. Yang, D. Chen, B. Lu, Q. Zhang et al., Structural regulation of P2-type layered oxide with anion/cation codoping strategy for sodium-ion batteries. Adv. Funct. Mater. **35**(14), 2418322 (2025). 10.1002/adfm.202418322

[17] Y. Lai, H. Xie, P. Li, B. Li, A. Zhao et al., Ion-migration mechanism: an overall understanding of anionic redox activity in metal oxide cathodes of Li/Na-ion batteries. Adv. Mater. **34**(47), 2206039 (2022). 10.1002/adma.202206039 10.1002/adma.202206039 36165216

[18] X. Cai, Z. Shadike, N. Wang et al., Constraining interlayer slipping in P2-type layered oxides with oxygen redox by constructing strong covalent bonds. J. Am. Chem. Soc. **147**(7), 5860–5870 (2025). 10.1021/jacs.4c14587 39908535 10.1021/jacs.4c14587

[19] J. Liu, W. Huang, R. Liu, J. Lang, Y. Li et al., Entropy tuning stabilizing P2-type layered cathodes for sodium-ion batteries. Adv. Funct. Mater. **34**(24), 2315437 (2024). 10.1002/adfm.202315437

[20] S. Yuan, L. Yu, G. Qian, Y. Xie, P. Guo et al., P2-type moisture-stable and high-voltage-tolerable cathodes for high-energy and long-life sodium-ion batteries. Nano Lett. **23**(5), 1743–1751 (2023). 10.1021/acs.nanolett.2c04465 36811529 10.1021/acs.nanolett.2c04465

[21] B. Peng, Z. Zhou, J. Shi, S. Xu, J. Yang et al., A customized strategy realizes stable cycle of large-capacity and high-voltage layered cathode for sodium-ion batteries. Angew. Chem. Int. Ed. **63**(50), e202411618 (2024). 10.1002/anie.202411618 10.1002/anie.202411618 39299916

[22] B. Peng, Y. Chen, L. Zhao, S. Zeng, G. Wan et al., Regulating the local chemical environment in layered O_3_^-^NaNi_0.5_Mn_0.5_O_2_ achieves practicable cathode for sodium-ion batteries. Energy Storage Mater. **56**, 631–641 (2023). 10.1016/j.ensm.2023.02.001

[23] Y. Wang, L. Wang, H. Zhu, J. Chu, Y. Fang et al., Ultralow-strain Zn-substituted layered oxide cathode with suppressed P2–O2 transition for stable sodium ion storage. Adv. Funct. Mater. **30**(13), 1910327 (2020). 10.1002/adfm.201910327

[24] Q. Shi, R. Qi, X. Feng, J. Wang, Y. Li et al., Niobium-doped layered cathode material for high-power and low-temperature sodium-ion batteries. Nat. Commun. **13**(1), 3205 (2022). 10.1038/s41467-022-30942-z 35680909 10.1038/s41467-022-30942-z PMC9184510

[25] K. Kubota, T. Asari, S. Komaba, Impact of Ti and Zn dual-substitution in P2 type Na_2/3_Ni_1/3_Mn_2/3_O_2_ on Ni–Mn and Na-vacancy ordering and electrochemical properties. Adv. Mater. **35**(26), 2300714 (2023). 10.1002/adma.202300714 10.1002/adma.202300714 37058281

[26] Q. Pei, M. Lu, Z. Liu, D. Li, X. Rao et al., Improving the Na_0.67_Ni_0.33_Mn_0.67_O_2_ cathode material for high-voltage cyclability via Ti/Cu codoping for sodium-ion batteries. ACS Appl. Energy Mater. **5**(2), 1953–1962 (2022). 10.1021/acsaem.1c03466

[27] G. Zhang, X. Yin, D. Ning, Y. Chai, R. Du et al., Crystal modulation of Mn-based layered oxide toward long-enduring anionic redox with fast kinetics for sodium-ion batteries. Angew. Chem. Int. Ed. **64**(3), e202415450 (2025). 10.1002/anie.202415450 10.1002/anie.202415450 39484729

[28] P. Zou, L. Yao, C. Wang, S.J. Lee, T. Li et al., Regulating cation interactions for zero-strain and high-voltage P2-type Na_2/3_Li_1/6_Co_1/6_Mn_2/3_O_2_ layered oxide cathodes of sodium-ion batteries. Angew. Chem. Int. Ed. **62**(28), e202304628 (2023). 10.1002/anie.202304628 10.1002/anie.202304628 37139583

[29] F. Ding, C. Zhao, D. Xiao, X. Rong, H. Wang et al., Using high-entropy configuration strategy to design Na-ion layered oxide cathodes with superior electrochemical performance and thermal stability. J. Am. Chem. Soc. **144**(18), 8286–8295 (2022). 10.1021/jacs.2c02353 35472274 10.1021/jacs.2c02353

[30] Z. Liu, R. Liu, S. Xu, J. Tian, J. Li et al., Achieving a deeply desodiated stabilized cathode material by the high entropy strategy for sodium-ion batteries. Angew. Chem. Int. Ed. **63**(29), e202405620 (2024). 10.1002/anie.202405620 10.1002/anie.202405620 38709194

[31] F. Ding, P. Ji, Z. Han, X. Hou, Y. Yang et al., Tailoring planar strain for robust structural stability in high-entropy layered sodium oxide cathode materials. Nat. Energy **9**(12), 1529–1539 (2024). 10.1038/s41560-024-01616-5

[32] Y. Huang, Y. Zhu, A. Nie, H. Fu, Z. Hu et al., Enabling anionic redox stability of P2-Na_5/6_Li_1/4_Mn_3/4_O_2_ by Mg substitution. Adv. Mater. **34**(9), 2105404 (2022). 10.1002/adma.202105404 10.1002/adma.202105404 34961966

[33] U. Maitra, R.A. House, J.W. Somerville, N. Tapia-Ruiz, J.G. Lozano et al., Oxygen redox chemistry without excess alkali-metal ions in Na_2/3_[Mg_0.28_Mn_0.72_]O_2_. Nat. Chem. **10**(3), 288–295 (2018). 10.1038/nchem.2923 29461536 10.1038/nchem.2923

[34] B. Peng, Y. Chen, F. Wang, Z. Sun, L. Zhao et al., Unusual site-selective doping in layered cathode strengthens electrostatic cohesion of alkali-metal layer for practicable sodium-ion full cell. Adv. Mater. **34**(6), 2103210 (2022). 10.1002/adma.202103210 10.1002/adma.202103210 34811831

[35] N. Ahmad, L. Yu, M.U. Muzaffar, B. Peng, Z. Tao et al., Dual-pillar effect in P2-type Na_0.67_Ni_0.33_Mn_0.67_O_2_ through Na site substitution achieve superior electrochemical and air/water dual-stability as cathode for sodium-ion batteries. Adv. Energy Mater. **15**(20), 2404093 (2025). 10.1002/aenm.202404093

[36] C. Zhao, Z. Yao, Q. Wang, H. Li, J. Wang et al., Revealing high Na-content P2-type layered oxides as advanced sodium-ion cathodes. J. Am. Chem. Soc. **142**(12), 5742–5750 (2020). 10.1021/jacs.9b13572 32118416 10.1021/jacs.9b13572 PMC7252945

[37] Y. Wang, R. Xiao, Y.-S. Hu, M. Avdeev, L. Chen, P2-Na_0.6_[Cr_0.6_Ti_0.4_]O_2_ cation-disordered electrode for high-rate symmetric rechargeable sodium-ion batteries. Nat. Commun. **6**, 6954 (2015). 10.1038/ncomms7954 25907679 10.1038/ncomms7954 PMC4421853

[38] P.-F. Wang, H.-R. Yao, X.-Y. Liu, Y.-X. Yin, J.-N. Zhang et al., Na(+)/vacancy disordering promises high-rate Na-ion batteries. Sci. Adv. **4**(3), eaar6018 (2018). 10.1126/sciadv.aar6018 29536049 10.1126/sciadv.aar6018 PMC5844706

[39] Y. Shi, P. Jiang, S. Wang, W. Chen, B. Wei et al., Slight compositional variation-induced structural disorder-to-order transition enables fast Na(+) storage in layered transition metal oxides. Nat. Commun. **13**(1), 7888 (2022). 10.1038/s41467-022-35597-4 36550128 10.1038/s41467-022-35597-4 PMC9780345

[40] J. Zhang, W. Wang, W. Wang, S. Wang, B. Li, Comprehensive review of P2-type Na_2/3_Ni_1/3_Mn_2/3_O_2_, a potential cathode for practical application of Na-ion batteries. ACS Appl. Mater. Interfaces **11**(25), 22051–22066 (2019). 10.1021/acsami.9b03937 31136141 10.1021/acsami.9b03937

[41] D.H. Lee, J. Xu, Y.S. Meng, An advanced cathode for Na-ion batteries with high rate and excellent structural stability. Phys. Chem. Chem. Phys. **15**(9), 3304–3312 (2013). 10.1039/C2CP44467D 23361584 10.1039/c2cp44467d

[42] A. Gutierrez, W.M. Dose, O. Borkiewicz, F. Guo, M. Avdeev et al., On disrupting the Na^+^-ion/vacancy ordering in P2-type sodium–manganese–nickel oxide cathodes for Na^+^-ion batteries. J. Phys. Chem. C **122**(41), 23251–23260 (2018). 10.1021/acs.jpcc.8b05537

[43] J.F. Qu, W. Wang, Y. Chen, G. Li, X.G. Li, Raman spectra study on nonstoichiometric compound Na_*x*_CoO_2_. Phys. Rev. B **73**(9), 092518 (2006). 10.1103/physrevb.73.092518

[44] W. Zhao, H. Kirie, A. Tanaka, M. Unno, S. Yamamoto et al., Synthesis of metal ion substituted P2-Na_2/3_Ni_1/3_Mn_2/3_O_2_ cathode material with enhanced performance for Na ion batteries. Mater. Lett. **135**, 131–134 (2014). 10.1016/j.matlet.2014.07.153

[45] C. Zhao, Y. Lu, L. Chen, Y.-S. Hu, Ni-based cathode materials for Na-ion batteries. Nano Res. **12**(9), 2018–2030 (2019). 10.1007/s12274-019-2451-3

[46] Y. Xiao, Y.-F. Zhu, H.-R. Yao, P.-F. Wang, X.-D. Zhang et al., A stable layered oxide cathode material for high-performance sodium-ion battery. Adv. Energy Mater. **9**(19), 1803978 (2019). 10.1002/aenm.201803978

[47] Y. Zhou, M. Pang, M. Zhang, Y. Yuan, Y. Yang et al., Controlling crystallographic orientation through composite phase regulation to unlock oxide cathode performance. Chem. Eng. J. **501**, 157527 (2024). 10.1016/j.cej.2024.157527

[48] K. Liu, S. Tan, X.-G. Sun, Q. Zhang, C. Li et al., Heteroatom anchoring to enhance electrochemical reversibility for high-voltage P2-type oxide cathodes of sodium-ion batteries. Nano Energy **128**, 109925 (2024). 10.1016/j.nanoen.2024.109925

[49] Z.-D. Liu, X.-W. Gao, J.-J. Mu, H. Chen, G. Gao et al., Multiphase riveting structure for high power and long lifespan potassium-ion batteries. Adv. Funct. Mater. **34**(26), 2315006 (2024). 10.1002/adfm.202315006

[50] T. Zhang, H. Ji, X. Hou, W. Ji, H. Fang et al., Promoting the performances of P2-type sodium layered cathode by inducing Na site rearrangement. Nano Energy **100**, 107482 (2022). 10.1016/j.nanoen.2022.107482

[51] Q. Zhao, F.K. Butt, M. Yang, Z. Guo, X. Yao et al., Tuning oxygen redox chemistry of P2-type manganese-based oxide cathode via dual Cu and Co substitution for sodium-ion batteries. Energy Storage Mater. **41**, 581–587 (2021). 10.1016/j.ensm.2021.06.029

[52] Y. Wang, J. Jin, X. Zhao, Q. Shen, X. Qu et al., Unexpected elevated working voltage by Na+/vacancy ordering and stabilized sodium-ion storage by transition-metal honeycomb ordering. Angew. Chem. Int. Ed. **63**(38), e202409152 (2024). 10.1002/anie.202409152 10.1002/anie.202409152 38923635

[53] Y. Fan, X. Ye, X. Yang, L. Guan, C. Chen et al., Zn/Ti/F synergetic-doped Na_0.67_Ni_0.33_Mn_0.67_O_2_ for sodium-ion batteries with high energy density. J. Mater. Chem. A **11**(7), 3608–3615 (2023). 10.1039/D2TA08315A

[54] X. Cai, N. Wang, L. Liang, X.-L. Li, R. Zhang et al., Fast oxygen redox kinetics induced by CoO_6_ octahedron with π-interaction in P2-type sodium oxides. Adv. Funct. Mater. **34**(51), 2409732 (2024). 10.1002/adfm.202409732

[55] J.G. Goiri, A. Van der Ven, Multishifter: software to generate structural models of extended two-dimensional defects in 3D and 2D crystals. Comput. Mater. Sci. **191**, 110310 (2021). 10.1016/j.commatsci.2021.110310

