# Supplementary material for: Regulation Engineering of Alkali Metal Interlayer Pillar in P2-Type Cathode for Ultra-High Rate and Long-Term Cycling Sodium-Ion Batteries
Source: Nanomicro Lett. 2026 Jan 8;18:105. doi: 10.1007/s40820-025-01918-7 (PMC12779844; doi:10.1007/s40820-025-01918-7)
Supplement: Supplementary file 1 — Supplementary file1 (DOCX 8763 kb) [file 40820_2025_1918_MOESM1_ESM.docx]

Supporting Information for

**Regulation Engineering of Alkali Metal Interlayer Pillar in P2-type Cathode for Ultra-high Rate and Long-term Cycling Sodium-Ion Batteries**

Xu Wang^1^, Zixiang Yang^1^, Yujia Cai^1^, Heng Ma^2^, Jinglei Xu^2^, Rabia Khatoon^3^, Zhizhen Ye^1^, Dashuai Wang^4,^ *, Muhammad Tariq Sajjad^3,^ *, Jianguo Lu^1,^ *

^1^State Key Laboratory of Silicon and Advanced Semiconductor Materials, School of Materials Science and Engineering, Zhejiang University, Hangzhou 310058, P. R. China

^2^Zhejiang HuaDian Electric Equipment Testing and Research Institute Co., Ltd., Hangzhou, 311100, P. R. China

^3^London South Bank University,103 Borough Road, London, SE1 0AA, England

^4^Institute of Zhejiang University-Quzhou, Quzhou 324000, P. R. China

*Corresponding authors. E-mail: [dswang@zju.edu.cn](mailto:dswang@zju.edu.cn) (Dashuai Wang); [sajjadt@lsbu.ac.uk](mailto:sajjadt@lsbu.ac.uk) (Muhammad Tariq Sajjad); [lujianguo@zju.edu.cn](mailto:lujianguo@zju.edu.cn) (Jianguo Lu)

**Supplementary Figures**

**
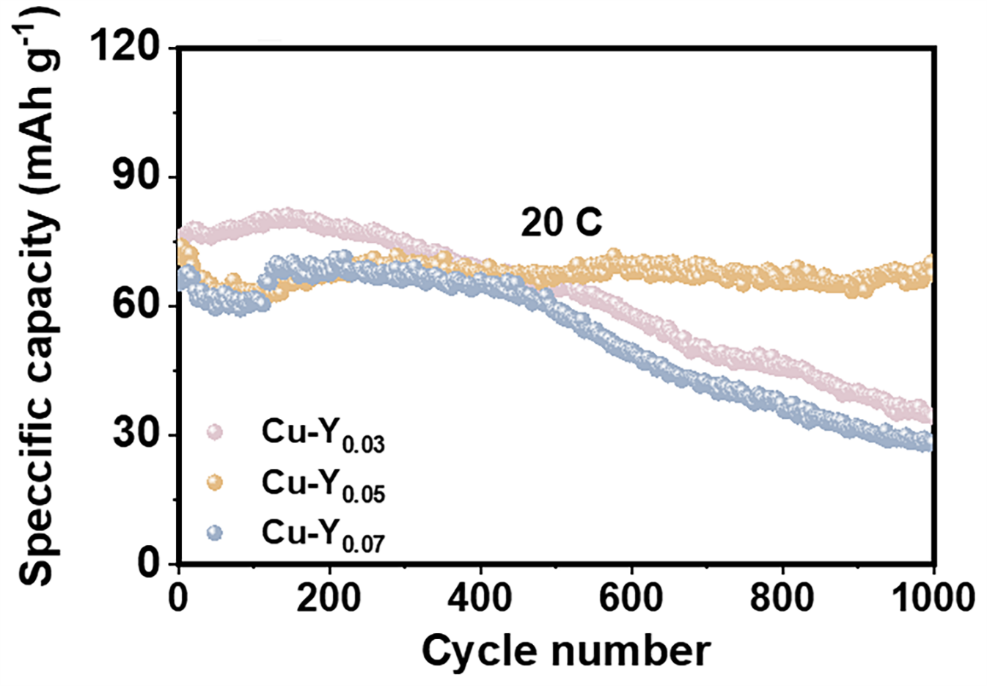
**

**Fig. S1** The long-term cycle performance comparison of Y_0.03_, Y_0.05_, and Y_0.07_ cathodes at an ultra-high rate of 20 C

To determine the optimal doping concentration of Y, a series of samples with varying Y gradient content were synthesized during the preliminary experimental stage. These included Na_0.67_Y_0.03_Ni_0.2_Cu_0.1_Mn_0.67_O_2_ (denoted as Y_0.03_), Na_0.67_Y_0.05_Ni_0.18_Cu_0.1_Mn_0.67_O_2_ (denoted as Y_0.05_), and Na_0.67_Y_0.07_Ni_0.16_Cu_0.1_Mn_0.67_O_2_ (denoted as Y_0.07_). As shown in Fig. S1, the Y_0.05_ sample exhibited the best capacity retention after 1000 cycles at a high current density. This result was used as the basis for subsequent experimental designs.

**
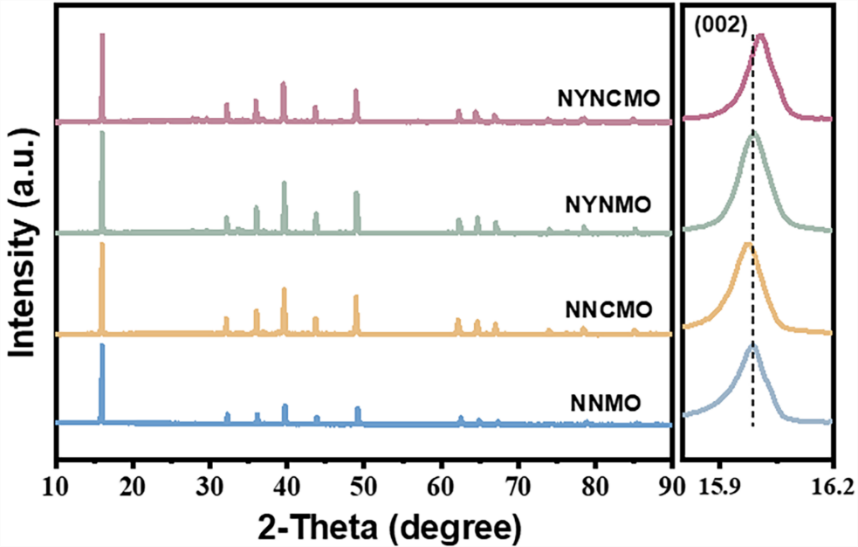
**

**Fig. S2** XRD patterns of the four materials

**
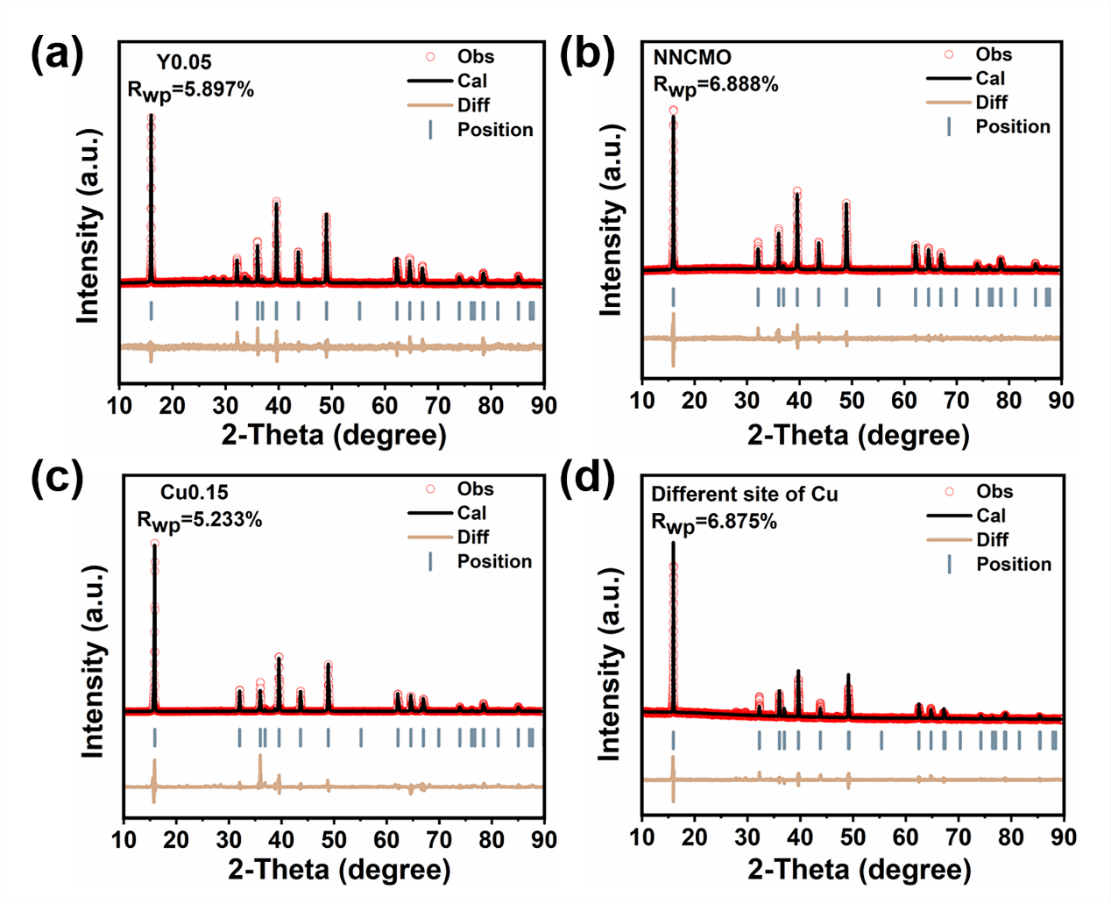
**

**Fig. S3** Rietveld refinement patterns of (**a**) NYNMO, (**b**) NNCMO, (**c**) NNC_0.15_MO, and (**d**) NCNYMO (putting Cu at the AM layer and the Y at the transition metal layer)


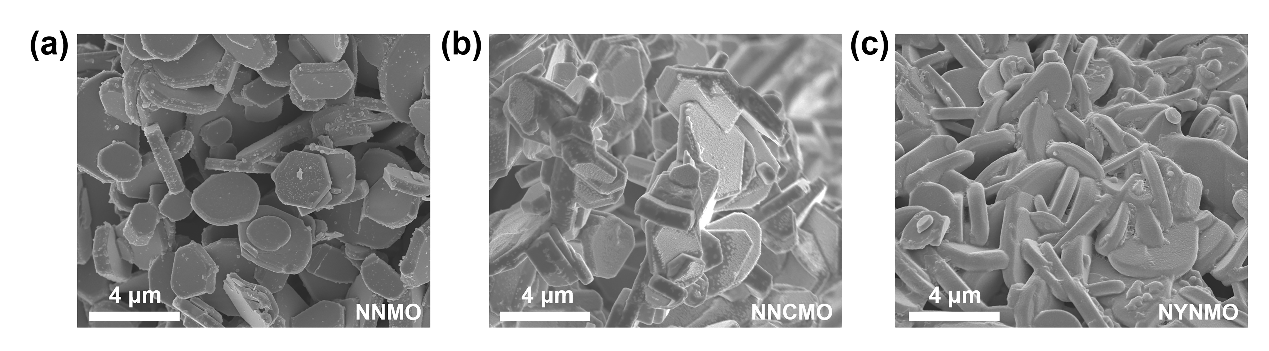


**Fig. S4** SEM image of (**a**) NNMO, (**b**) NNCMO, and (**c**) NYNMO

**
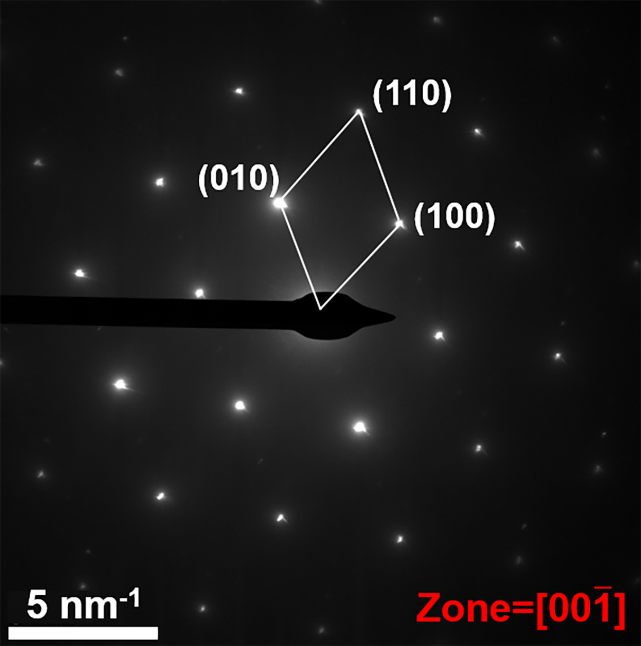
**

**Fig. S5** SAED pattern collected from [00$\bar{\text{1}}$] axis
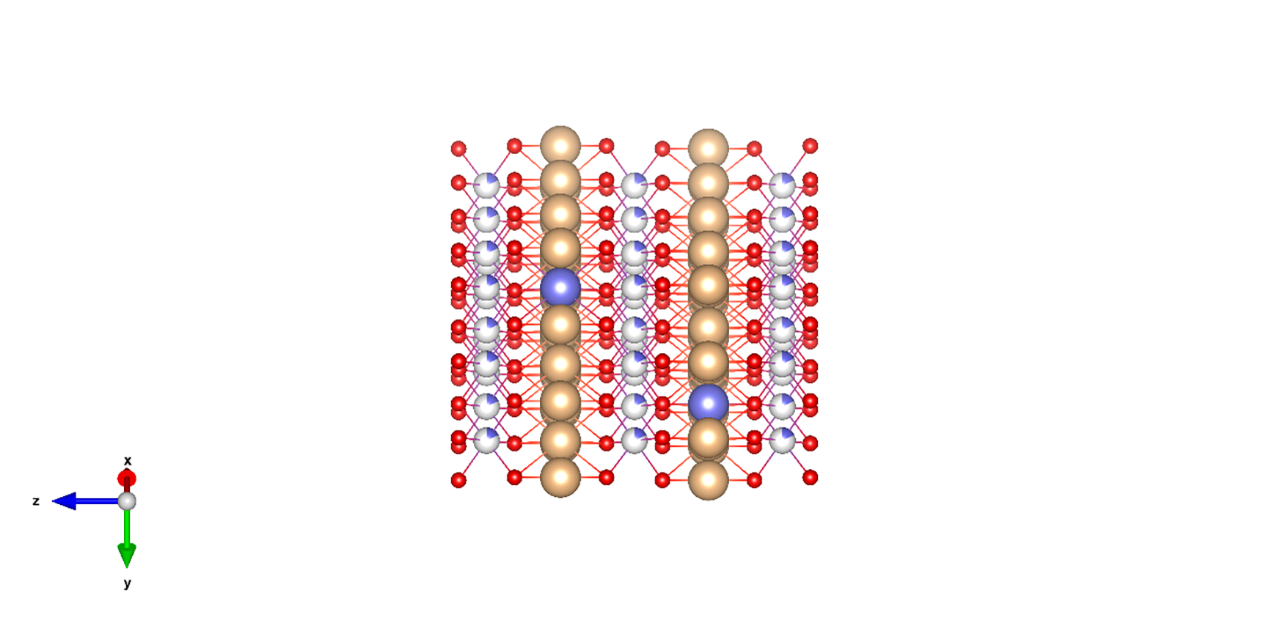


**Fig. S6** Crystal structure viewed from [$\bar{\text{2}}\bar{\text{1}}\text{0]}$ axis (the red spheres, white spheres, violet spheres, and dark yellow spheres represent O ions, metal ions (Ni, Mn, Cu), Y ions, and Na ions, respectively)


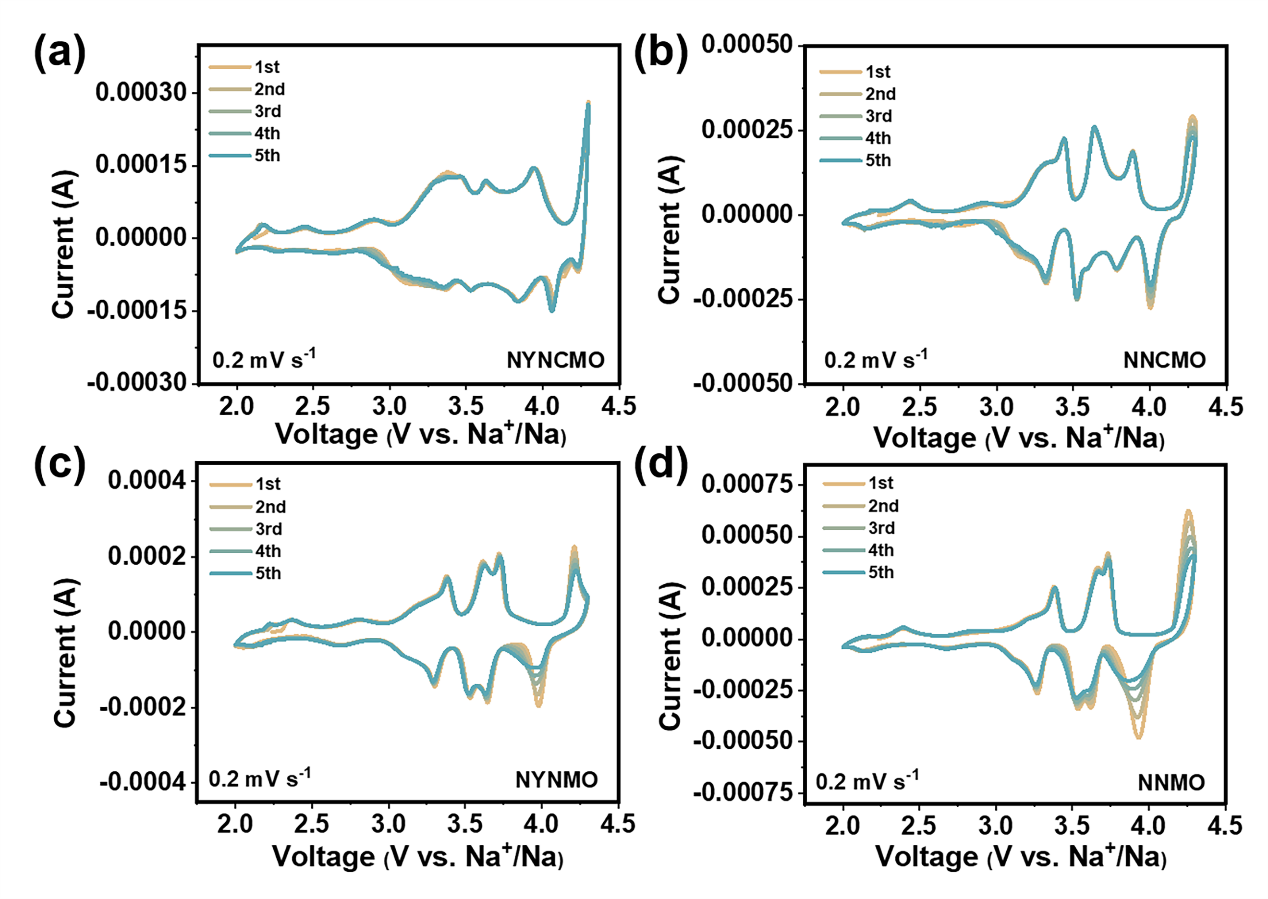


**Fig. S7** The first five cycles CVs of (**a**) NYNCMO, (**b**) NNCMO, (**c**) NYNMO and (**d**) NNMO at a scanning speed of 0.2 mV s^-1^


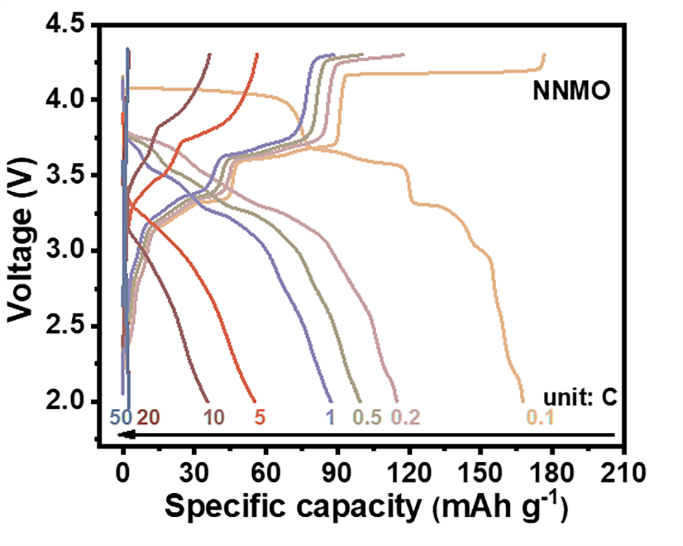


**Fig. S8** Charge and discharge curves of NNMO electrode materials at different rates


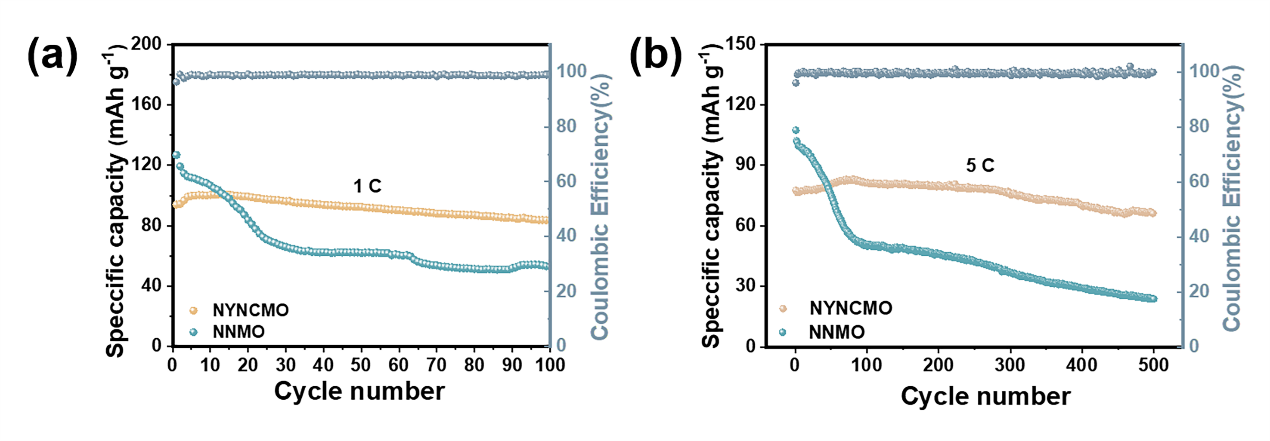


**Fig. S9** The comparison of cycling performances of NNMO and NYNCMO at (**a**) 1 C and (**b**) 5 C


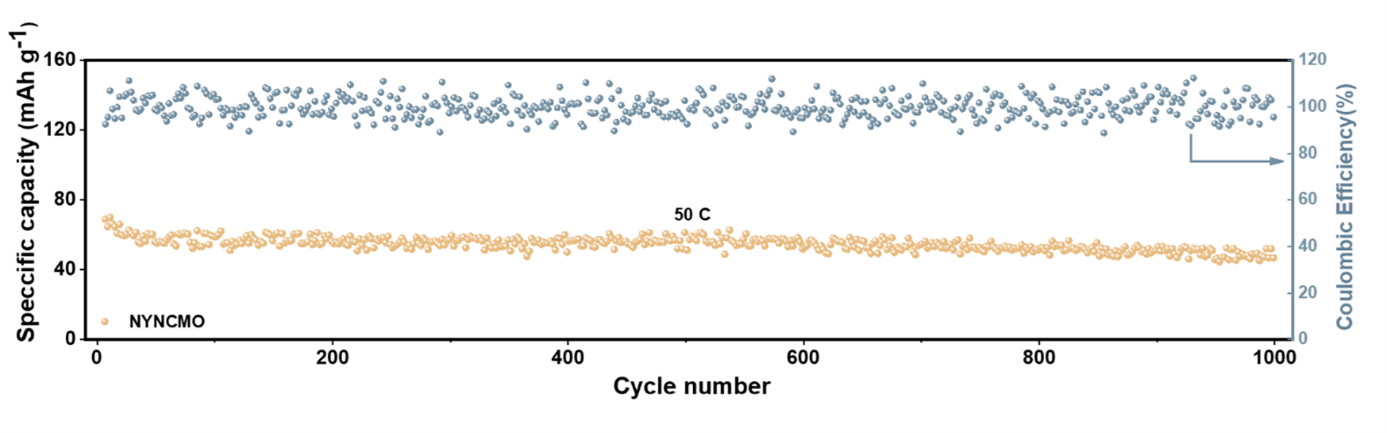


**Fig. S10** The cycling performances of NYNCMO at an ultra-high rate of 50 C


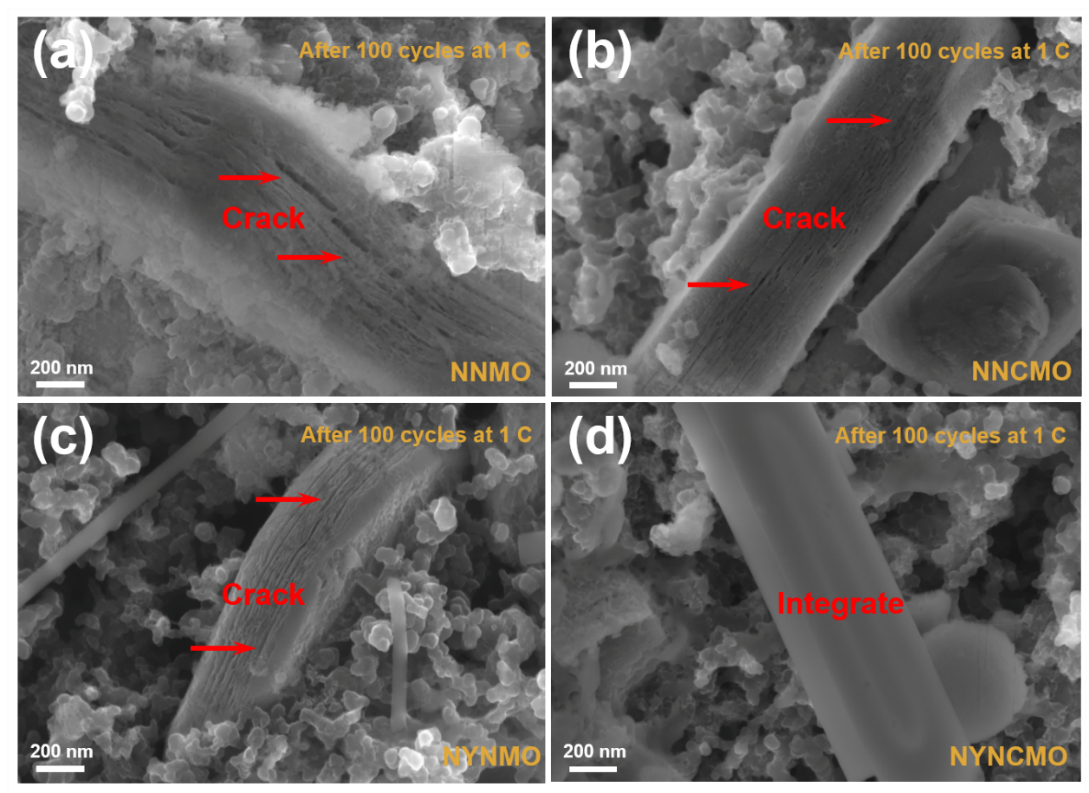


**Fig. S11** Structural characterizations of electrode after deep cycling: SEM image after cycling 100 cycles at 1 C of (**a**) NNMO, (**b**) NNCMO, (**c**) NYNMO and (**d**) NYNCMO


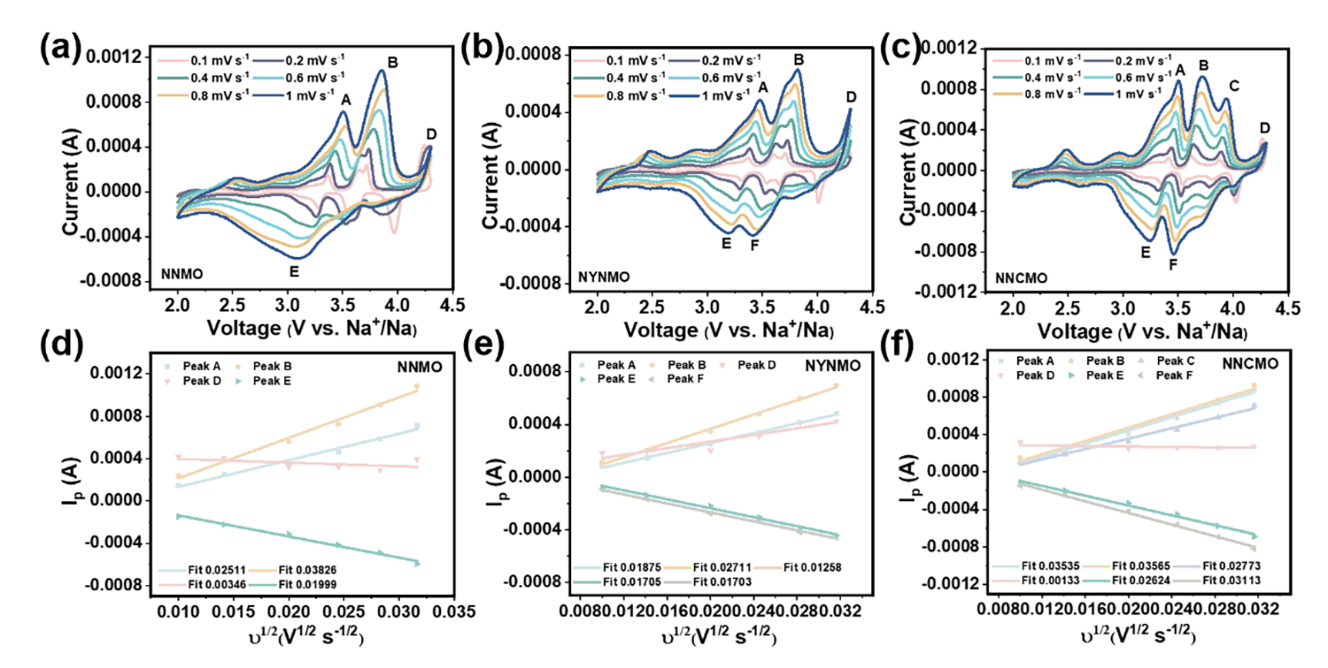


**Fig. S12** Multisweep CVs of (**a**) NNMO, (**b**) NYNMO and (**c**) NNCMO at various scan rates and (**d-f**) the corresponding cycling response of different peaks of NNMO, NYNMO and NNCMO, respectively


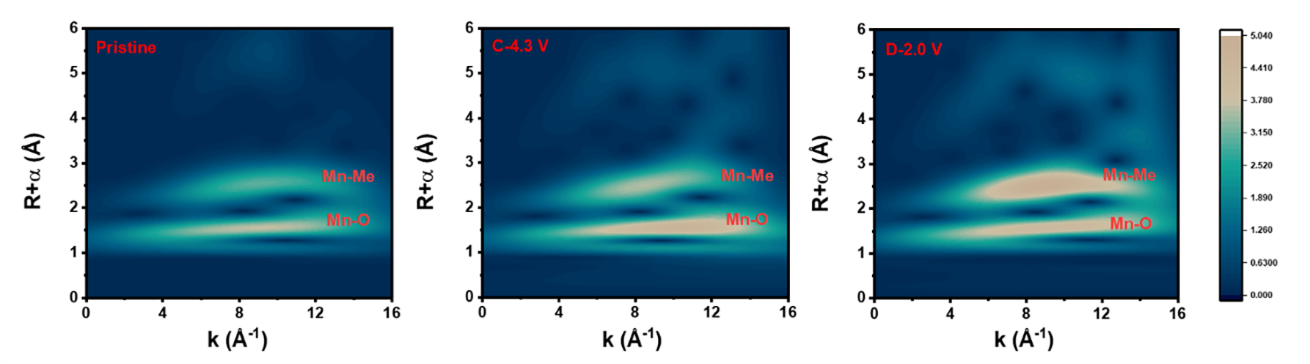


**Fig. S13** Wavelet transform (WT) contour plots of Mn in NYNCMO-pristine, charge to 4.3 V, and discharge to 2.0 V

**
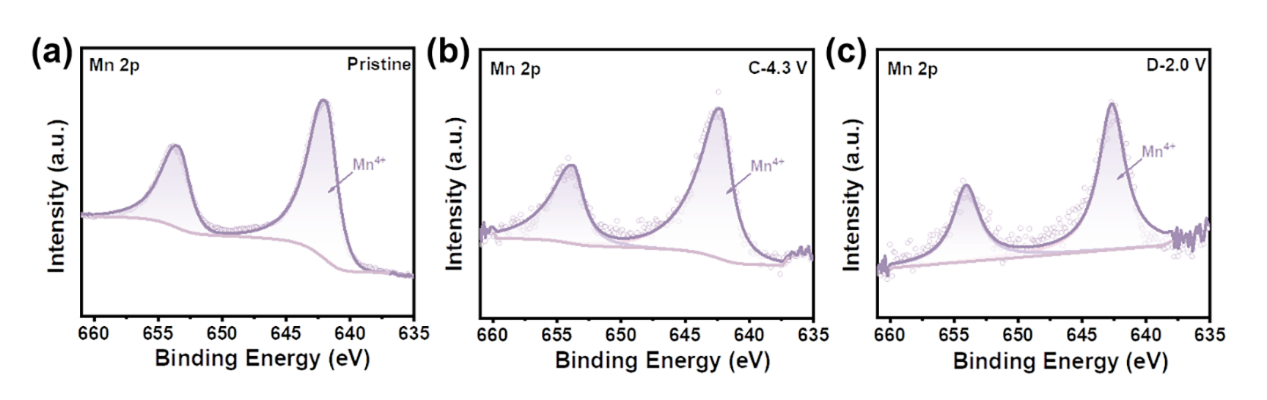
**

**Fig. S14** Ex-situ X-ray photoelectron spectroscopy (XPS) of Mn high-resolution spectra analysis at different states of NYNCMO (**a**) Pristine; (**b**) Charge-4.3 V; (**c**) Discharge-2.0 V

**
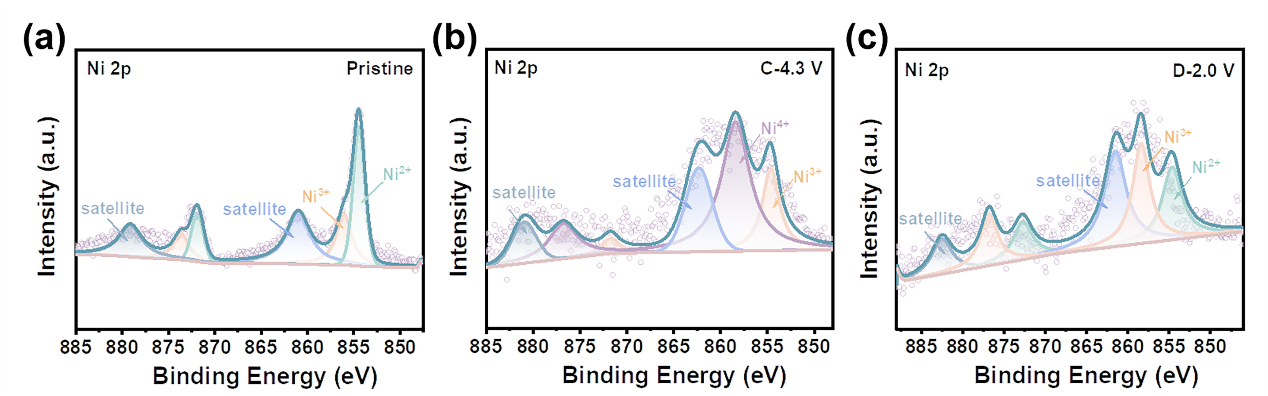
**

**Fig. S15** Ex-situ X-ray photoelectron spectroscopy (XPS) of Ni high-resolution spectra analysis at different states of NYNCMO (**a**) Pristine; (**b**) Charge-4.3 V; (**c**) Discharge-2.0 V

**
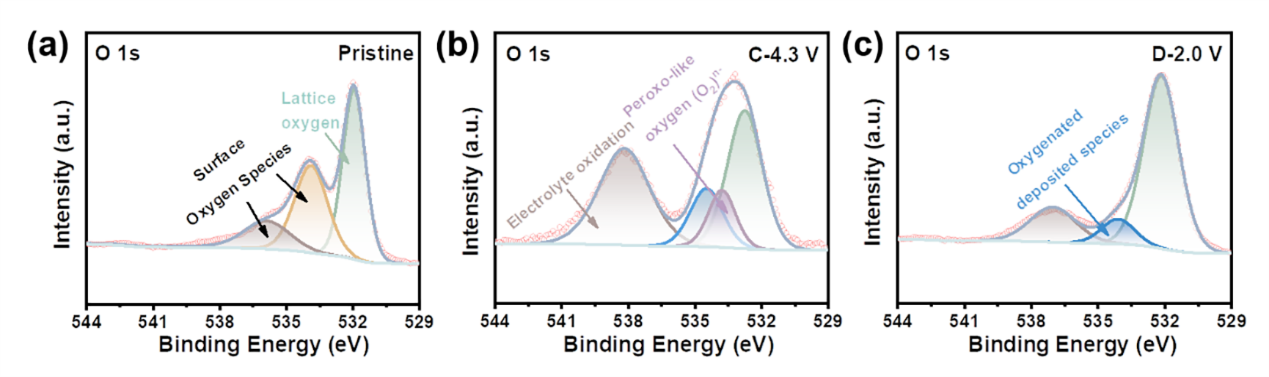
**

**Fig. S16** Ex-situ X-ray photoelectron spectroscopy (XPS) of O high-resolution spectra analysis at different states of NYNCMO (**a**) Pristine; (**b**) Charge-4.3 V; (**c**) Discharge-2.0 V

**
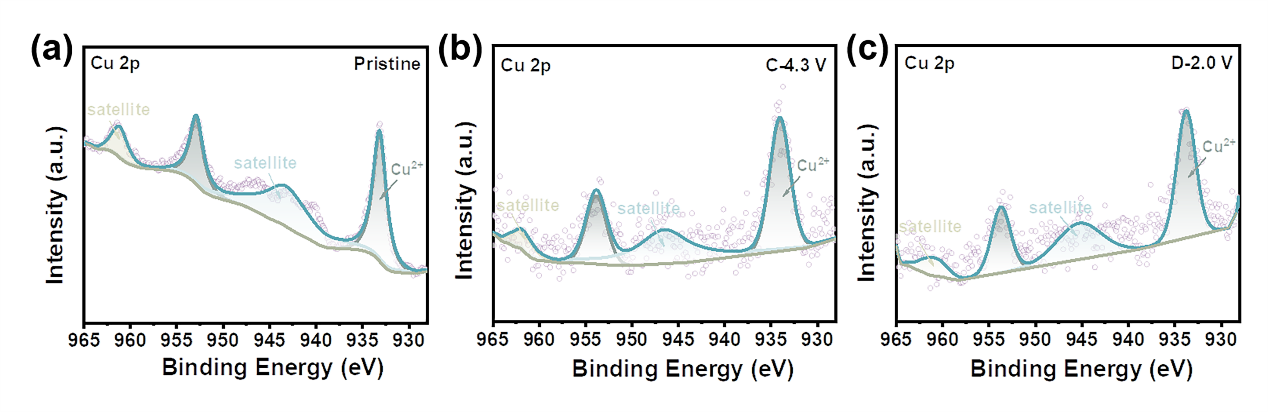
**

**Fig. S17** Ex-situ X-ray photoelectron spectroscopy (XPS) of Cu high-resolution spectra analysis at different states of NYNCMO (**a**) Pristine; (**b**) Charge-4.3 V; (**c**) Discharge-2.0 V

**
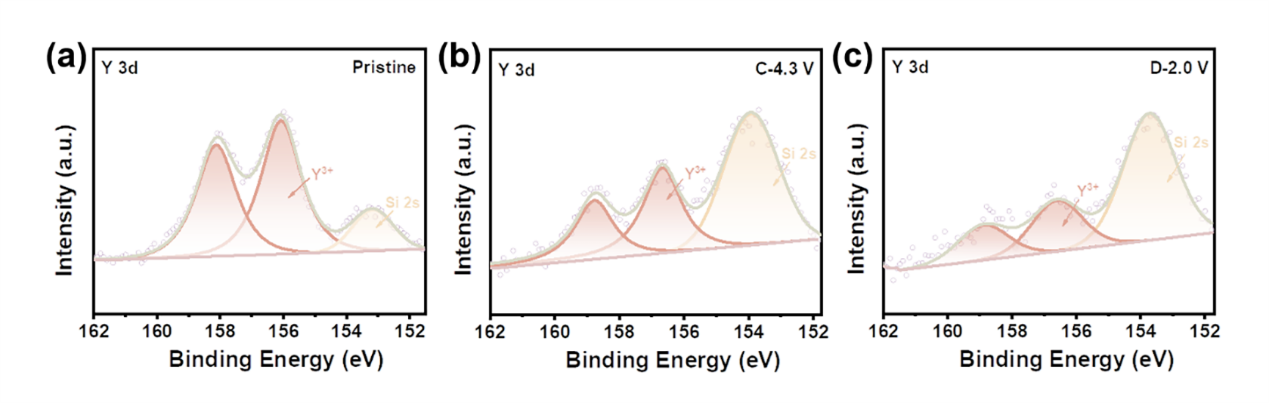
**

**Fig. S18** Ex-situ X-ray photoelectron spectroscopy (XPS) of Y high-resolution spectra analysis at different states of NYNCMO (**a**) Pristine; (**b**) Charge-4.3 V; (**c**) Discharge-2.0 V


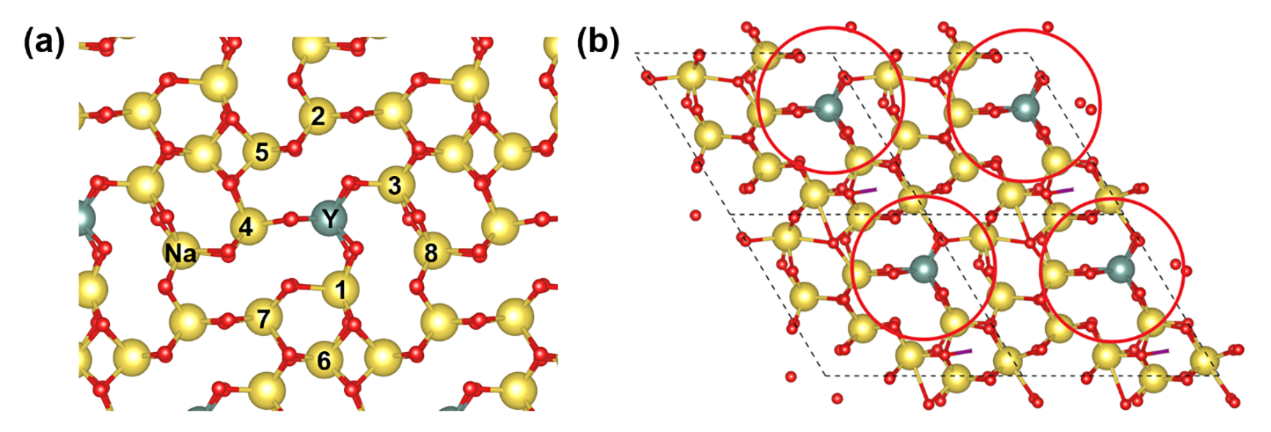


**Fig. S19** Diagram of selected 8 Na sites adjacent to Y for the calculation of bond length


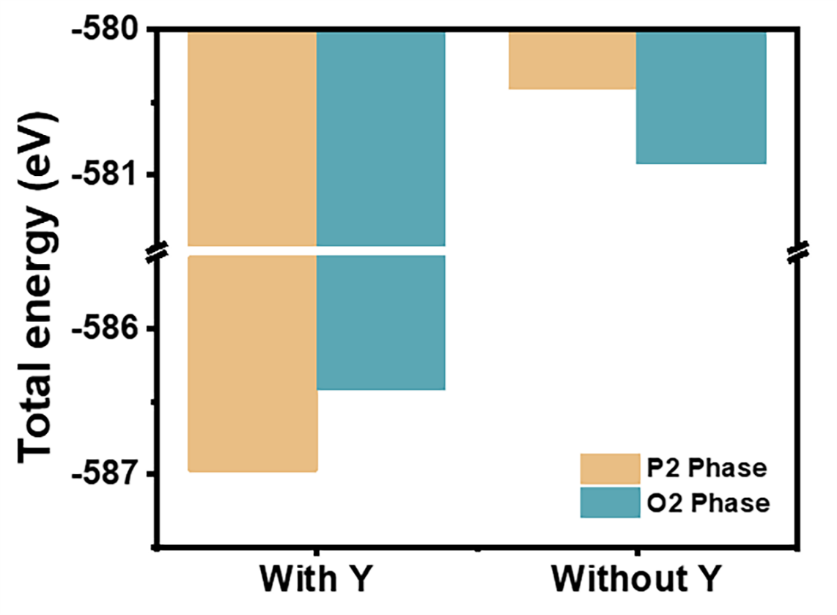


**Fig. S20** Comparison of total energy between P2 phase and O2 phase with and without Y


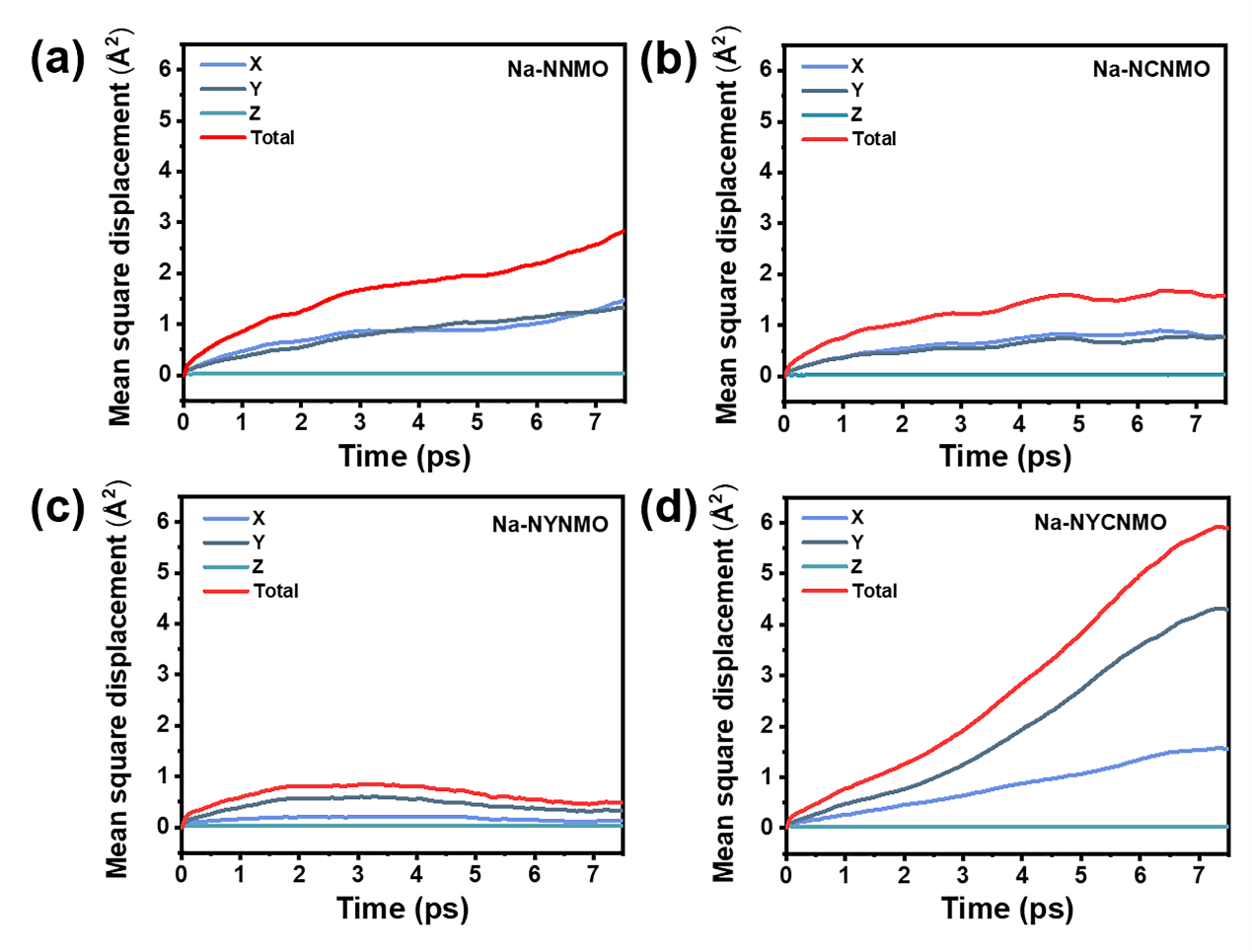


**Fig. S21** Specific mean square displacement performance of Na ions in (**a**) NNMO, (**b**) NNCMO, (**c**) NYNMO, and (**d**) NYNCMO


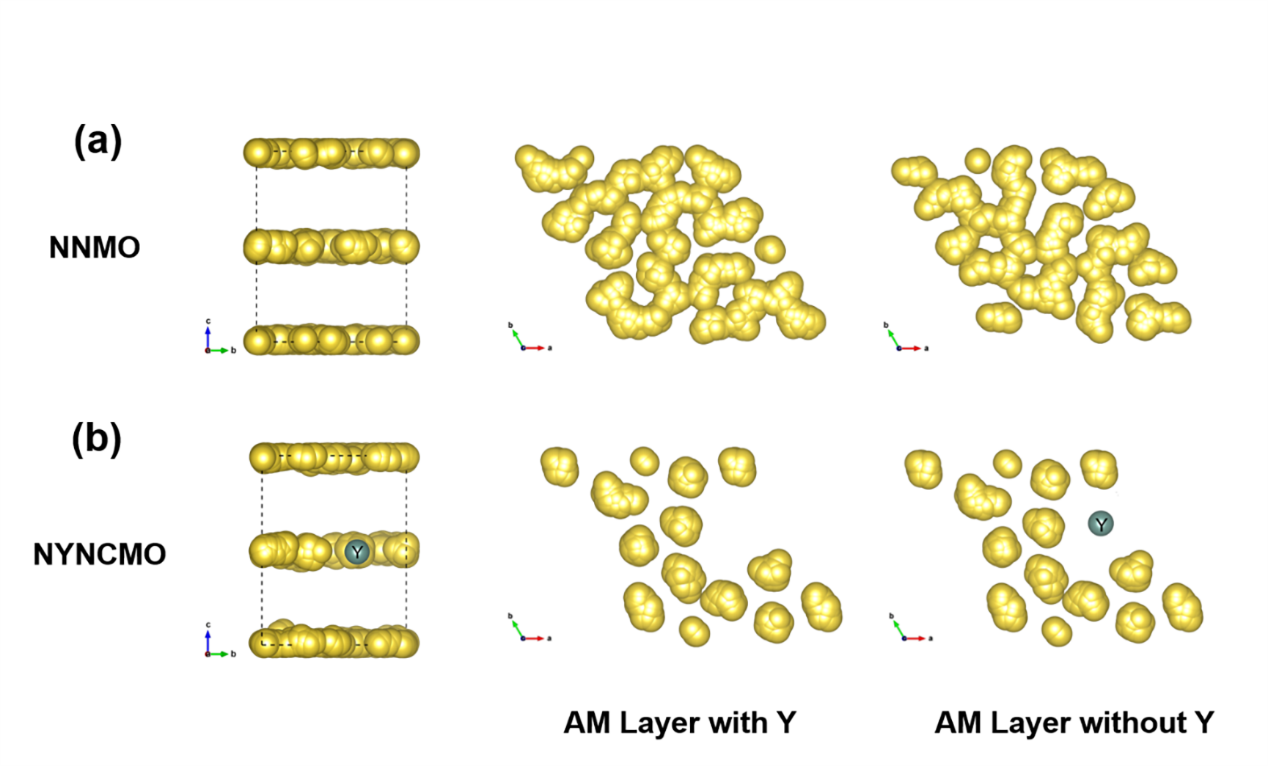


**Fig. S22** Schematic diagram of Na^+^ diffusion trajectories in (**a**) NNMO and (**b**) NYNCMO


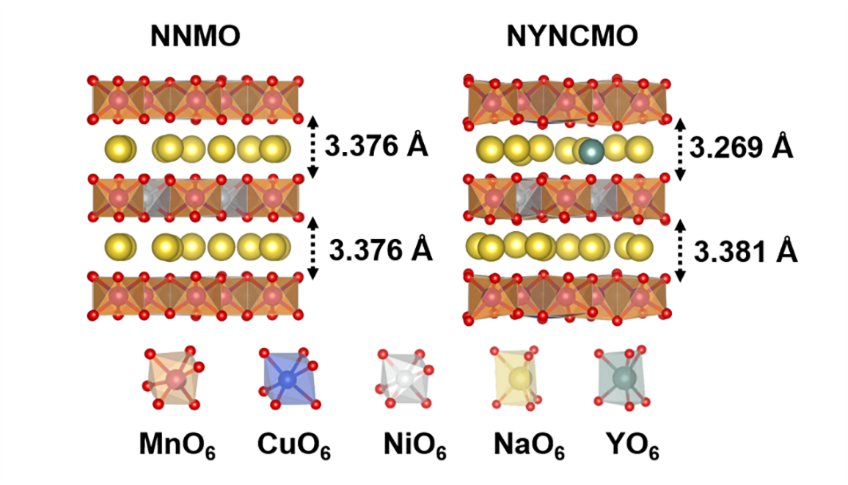


**Fig. S23** Schematic diagram of interlayer spacing variation between NNMO and NYNCMO

**Supplementary Tables**

**Table S1** Inductively coupled plasma optical emission spectrometry (ICP-OES) of NYNCMO

|  | **Na** | **Y** | **Ni** | **Cu** | **Mn** |
| --- | --- | --- | --- | --- | --- |
| **NYNCMO** | 0.63 | 0.04 | 0.18 | 0.10 | 0.68 |

**Table S2** Crystallographic parameters of NYNCMO obtained from XRD Rietveld refinement

| Space group | P63/mmc |  |  |  |  |
| --- | --- | --- | --- | --- | --- |
| atom | site | x | y | z | Occ. |
| Na_f_ | 2b | 0 | 0 | 0.25 | 0.33 |
| Na_e_ | 2d | 0.3333 | 0.6667 | 0.25 | 0.33 |
| Y | 2d | 0.3333 | 0.6667 | 0.25 | 0.05 |
| Ni | 2a | 0 | 0 | 0 | 0.18 |
| Cu | 2a | 0 | 0 | 0 | 0.1 |
| Mn | 2a | 0 | 0 | 0 | 0.67 |
| O | 4f | 0.3333 | 0.6667 | 0.0945 | 1.0 |
| a=2.8933 Å | c=11.1586 Å | V=80.899 Å^3^ | R_w_=5.769% | Gof=2.18 |  |

**Table S3** Crystallographic parameters of NYNMO obtained from XRD Rietveld refinement

| Space group | P63/mmc |  |  |  |  |
| --- | --- | --- | --- | --- | --- |
| atom | site | x | y | z | Occ. |
| Na_f_ | 2b | 0 | 0 | 0.25 | 0.33 |
| Na_e_ | 2d | 0.3333 | 0.6667 | 0.25 | 0.33 |
| Ni | 2a | 0 | 0 | 0 | 0.28 |
| Mn | 2a | 0 | 0 | 0 | 0.67 |
| Y | 2a | 0 | 0 | 0 | 0.05 |
| O | 4f | 0.3333 | 0.6667 | 0.0945 | 1.0 |
| a=2.8854 Å | c=11.1651 Å | V=80.501 Å^3^ | R_w_=5.897% | Gof=2.20 |  |

**Table S4** Crystallographic parameters of NNC_0.1_MO obtained from XRD Rietveld refinement

| Space group | P63/mmc |  |  |  |  |
| --- | --- | --- | --- | --- | --- |
| atom | site | x | y | z | Occ. |
| Na_f_ | 2b | 0 | 0 | 0.25 | 0.2820 |
| Na_e_ | 2d | 0.3333 | 0.6667 | 0.25 | 0.3880 |
| Ni | 2a | 0 | 0 | 0 | 0.23 |
| Cu | 2a | 0 | 0 | 0 | 0.10 |
| Mn | 2a | 0 | 0 | 0 | 0.67 |
| O | 4f | 0.3333 | 0.6667 | 0.0945 | 1.0 |
| a=2.8867 Å | c=11.1907 Å | V=80.759 Å^3^ | R_w_=6.888% | Gof=2.56 |  |

**Table S5** Crystallographic parameters of NNC_0.15_MO obtained from XRD Rietveld refinement

| Space group | P63/mmc |  |  |  |  |
| --- | --- | --- | --- | --- | --- |
| atom | site | x | y | z | Occ. |
| Na_f_ | 2b | 0 | 0 | 0.25 | 0.3729 |
| Na_e_ | 2d | 0.3333 | 0.6667 | 0.25 | 0.3419 |
| Ni | 2a | 0 | 0 | 0 | 0.18 |
| Cu | 2a | 0 | 0 | 0 | 0.15 |
| Mn | 2a | 0 | 0 | 0 | 0.67 |
| O | 4f | 0.3333 | 0.6667 | 0.0945 | 1.0 |
| a=2.8882 Å | c=11.1909 Å | V=80.843 Å^3^ | R_w_=5.233% | Gof=2.70 |  |

**Table S6** The agreement factors variation after putting Cu ion at the Na sites

| Space group | P63/mmc |  |  |  |  |
| --- | --- | --- | --- | --- | --- |
| atom | site | x | y | z | Occ. |
| Na_f_ | 2b | 0 | 0 | 0.25 | 0.33 |
| Na_e_ | 2d | 0.3333 | 0.6667 | 0.25 | 0.33 |
| Cu1 | 2a | 0 | 0 | 0 | 0.05 |
| Ni | 2a | 0 | 0 | 0 | 0.18 |
| Mn | 2a | 0 | 0 | 0 | 0.67 |
| Cu2 | 2a | 0 | 0 | 0 | 0.05 |
| Y | 2a | 0 | 0 | 0 | 0.05 |
| O | 4f | 0.3333 | 0.6667 | 0.0945 | 1.0 |
| a=2.8753 Å | c=11.0984 Å | V=79.462 Å^3^ | R_w_=6.875% | Gof=2.82 |  |

**Table S7** Crystallographic parameters of NNMO obtained from XRD Rietveld refinement

| Space group | P63/mmc |  |  |  |  |
| --- | --- | --- | --- | --- | --- |
| atom | site | x | y | z | Occ. |
| Na_f_ | 2b | 0 | 0 | 0.25 | 0.33 |
| Na_e_ | 2d | 0.3333 | 0.6667 | 0.25 | 0.33 |
| Ni | 2a | 0 | 0 | 0 | 0.28 |
| Mn | 2a | 0 | 0 | 0 | 0.67 |
| O | 4f | 0.3333 | 0.6667 | 0.0945 | 1.0 |
| a=2.8713 Å | c=11.1090 Å | V=79.317 Å^3^ | R_w_=4.988% | Gof=2.10 |  |

**Table S8** Comparison of electrochemical properties between NYNCMO and other reported values in half battery

| Samples | Max Current rate (A g^-1^)(Rate) | Max cycle numbers | | Retention(%) |
| --- | --- | --- | --- | --- |
| P2-Na_7/9_Li_1/9_Ni_2/9_Mn_5/9_Ti_1/9_O_2_ | 1.5 (10 C) | 200 | 88 | |
| Na_0.67_Ni_0.28_Zn_0.05_Mn_0.62_Ti_0.05_O_1.95_F_0.05_ | 1.7 (10 C) | 1000 | 86 | |
| Na_0.67_Ni_0.23_Mg_0.1_Mn_0.65_Sn_0.02_O_2_ | 1 | 500 (0.5 A g^-1^) | 80 | |
| P2-Na_0.8_Cu_0.22_Li_0.08_Mn_0.67_O_2_ | 6.5 (50 C) | 500 (0.65 A g^-1^) | 86.5 | |
| Na_0.67_Mn_0.6_Ni_0.2_Cu_0.1_Co_0.1_O_2_ | 2 (20 C) | 500 (1 A g^-1^) | 82.07 | |
| Na_0.72_Li_0.24_Mn_0.76_O_2_ | 2 (10 C) | 300 (1 A g^-1^) | 84.28 | |
| Na_0.67_Ni_0.23_Mg_0.05_Cu_0.05_Mn_0.67_O_2_ | 1.7 (10 C) | 2000 | 83.4 | |
| Na_0.9_[Ni_0.3_Mn_0.55_Cu_0.1_Ti_0.05_]O_2_ | 0.75 | 600 (0.3 A g^-1^ ) | 83.4 | |
| Na_0.6_Mn_0.93_Fe_0.04_Mg_0.03_O_2_ | 3 (10 C) | 1000 (1 A g^-1^) | 72.5 | |
| Na_2/3_Ni_1/6_Mn_2/3_Cu_1/9_Mg_1/18_O_2_ | 3.6 (30 C) | 500 (1 A g^-1^) | 84.2 | |
| **This Work** | 3.0 (20 C) | 1600 | 71.7 | |
|  | 7.5 (50 C) | 1000 | 76 | |
|  | 1.5 (10 C) | 3000 | 65 | |

**Table S9** Comparison of EIS fitting results of four materials

|  | NNMO | NYNMO | NNCMO | NYNCMO |
| --- | --- | --- | --- | --- |
| Rs/Ω | 4.046 | 5.584 | 7.606 | 4.001 |
| Rct/Ω | 784.7 | 342.2 | 526 | 222.8 |

**Table S10** The diffusion coefficient in X, Y, Z direction and total of the four materials

| Diffusion Coefficient | X direction (cm^2^/s) | Y direction (cm^2^/s) | Z direction (cm^2^/s) | Average (cm^2^/s) |
| --- | --- | --- | --- | --- |
| NNMO | 0.7447×10^-5^ | 0.9563×10^-5^ | 0.1122×10^-7^ | 0.5674×10^-5^ |
| NYNMO | 0.9805×10^-6^ | 0.2905×10^-5^ | 0.3244×10^-7^ | 0.1306×10^-5^ |
| NNCMO | 0.6708×10^-5^ | 0.5618×10^-5^ | 0.1615×10^-7^ | 0.4114×10^-5^ |
| NYNCMO | 0.1012×10^-4^ | 0.2531×10^-4^ | 0.2299×10^-7^ | 0.1182×10^-4^ |

**Table S11** The ionic mobility in X, Y, Z direction and total of the four materials

| Ionic Mobility | X direction (cm^2^/s/V) | Y direction (cm^2^/s/V) | Z direction (cm^2^/s/V) | Average (cm^2^/s/V) |
| --- | --- | --- | --- | --- |
| NNMO | 0.1235×10^-3^ | 0.1585×10^-3^ | 0.1860×10^-6^ | 0.9406×10^-4^ |
| NYNMO | 0.1625×10^-4^ | 0.4815×10^-4^ | 0.5378×10^-6^ | 0.2165×10^-4^ |
| NNCMO | 0.1112×10^-3^ | 0.9313×10^-4^ | 0.2677×10^-6^ | 0.6820×10^-4^ |
| NYNCMO | 0.1678×10^-3^ | 0.4196×10^-3^ | 0.3182×10^-6^ | 0.1959×10^-3^ |
